# Combining X-ray and NMR Crystallography to Explore the Crystallographic Disorder in Salbutamol Oxalate

**DOI:** 10.1021/acs.cgd.1c01093

**Published:** 2022-07-20

**Authors:** Aneesa J. Al-Ani, Patrick M. J. Szell, Zainab Rehman, Helen Blade, Helen P. Wheatcroft, Leslie P. Hughes, Steven P. Brown, Chick C. Wilson

**Affiliations:** † Centre for Sustainable and Circular Technologies (CSCT), University of Bath, Claverton Down, Bath BA2 7AY, U.K.; ‡ Department of Physics, University of Warwick, Coventry CV4 7AL, U.K.; § Oral Product Development, Pharmaceutical Technology & Development, Operations, AstraZeneca, Macclesfield SK10 2NA, U.K.; ∥ Chemical Development, Pharmaceutical Technology & Development, Operations, AstraZeneca, Macclesfield SK10 2NA, U.K.

## Abstract

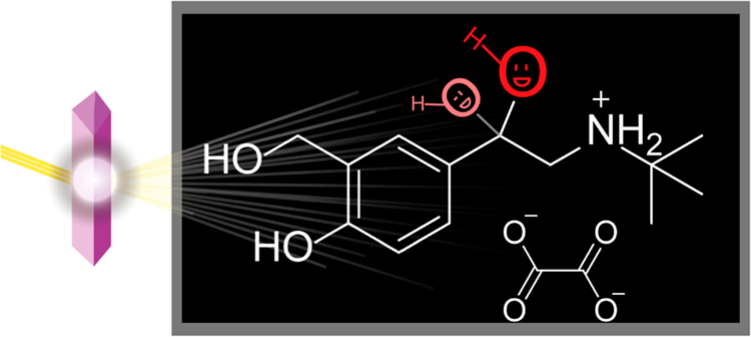

Salbutamol is an active pharmaceutical ingredient commonly used to treat respiratory distress and is listed by the World Health Organization as an essential medicine. Here, we establish the crystal structure of its oxalate form, salbutamol oxalate, and explore the nature of its crystallographic disorder by combined X-ray crystallography and ^13^C cross-polarization (CP) magic-angle spinning (MAS) solid-state NMR. The *C–OH chiral center of salbutamol (note that the crystal structures are a racemic mixture of the two enantiomers of salbutamol) is disordered over two positions, and the *tert*-butyl group is rotating rapidly, as revealed by ^13^C solid-state NMR. The impact of crystallization conditions on the disorder was investigated, finding variations in the occupancy ratio of the *C–OH chiral center between single crystals and a consistency across samples in the bulk powder. Overall, this work highlights the contrast between investigating crystallographic disorder by X-ray diffraction and solid-state NMR experiment, and gauge-including projector-augmented-wave (GIPAW) density functional theory (DFT) calculations, with their combined use, yielding an improved understanding of the nature of the crystallographic disorder between the local (i.e., as viewed by NMR) and longer-range periodic (i.e., as viewed by diffraction) scale.

## Abstract 2

The crystal structure of salbutamol oxalate, a new salt form of salbutamol examined by NMR crystallography, reveals the crystallographic disorder at the *C−OH chiral center.

## Introduction

Crystallization is an essential step in the manufacture of many pharmaceutical solids, with the majority of active pharmaceutical ingredients (APIs) marketed in their solid form.^1,2^ Controlling the crystallization process allows for the selection of the solid form with the most suitable physicochemical properties and can help ensure uniformity and phase purity.^2−4^ A lack of characterization of an API’s crystalline form can have devastating consequences, such as unexpected changes to the performance and inconsistent physicochemical properties (e.g., compressibility and dissolution).^5−7^

Crystallographic disorder occurring in crystalline pharmaceuticals is a poorly understood solid-state behavior, and it can be difficult to characterize or control. Further, disorder can impact the physicochemical properties of a pharmaceutical, such as stability, and can lead to difficulties in the crystallization and manufacturing process.^8,9^ The challenge arises in part due to a lack of analytical tools available as current characterization methods have difficulty in distinguishing subtle changes in the crystallographic disorder in organic systems.^9^

The two most common types of crystallographic disorder observed in pharmaceutical materials are orientational (disarrangement of a whole molecule) and conformational disorder (disarrangement of part of a molecule).^9^ This leads to the formation of materials with freedom of movement around multiple positions, and this movement cannot be resolved by conventional X-ray diffraction (XRD). Rather, the structure is refined from the Bragg intensities which show an average picture of all the positions. The presence of molecular disorder in a solid form can increase the Gibbs free energy of a bulk material and can give rise to weaker molecular packing and increased molecular motions.^9^ The variation can occur in a single unit cell via molecular mobility (dynamic disorder) or can be distributed among several unit cells (static disorder).^10^ In addition to the difficulty in resolving the disorder, there is a common misconception that every single crystal in a batch is identical, which is an oversimplified view. The analysis of one single crystal does not provide information about the overall arrangement of molecules in the bulk and thus provides a limited picture of the molecular disorder; it may not be a fully representative characterization method. The development of new methodologies to overcome this limitation is required to gain a detailed picture of the molecular disorder and the identification of subtle differences between crystals.

Various bulk analytical methods are currently used to characterize the disorder. The majority of these methods characterize a solid at the surface or particulate level such as isothermal micro-calorimetry,^11^ dynamic vapor sorption,^12,13^ atomic force microscopy,^14,15^ and transmission electron microscopy.^16^ The characterization of disorder at the molecular level in the bulk is currently limited to powder XRD^17,18^ and some spectroscopic methods such as solid-state NMR,^19−29^ Raman,^30^ and dielectric spectroscopy.^31^ Single crystal XRD is a fundamental method in crystallography that provides detailed information about the internal lattice of a crystalline material and the nature of the disorder;^32−34^ however, it is not a bulk analysis method.^35^ The ability to distinguish subtle changes in disorder at the molecular level via single crystal XRD (SXRD) remains a challenge and it is limited to a generalized average value from one single crystal with no clear way to quantify the range of the disorder occurring in the many crystals in the bulk powder.

Atomic displacement parameters (ADPs) from X-ray data often provide the first indication that a structure is disordered especially in the case of static disorder, but identifying dynamic disorder is particularly challenging. Variable-temperature X-ray measurements can provide additional insights, but the SXRD data are usually only collected at one specific temperature, with a low temperature, between 100 and 150 K, usually preferred. Solid-state NMR, especially when combined with density functional theory (DFT) calculations, provides the opportunity to derive information about the nature of any disorder using variable-temperature experiments and relaxation time measurements. Solid-state NMR is sensitive to short-range order, and with experimental timescales (for the radiofrequency pulses and acquisition of the time-domain signal) ranging from microseconds to tens of milliseconds, the distinction between static and dynamic disorder is possible. Other techniques such as Terahertz time-domain spectroscopy^36^ are emerging for probing the sorts of motions that NMR may not be sensitive to. A combination of complementary characterization techniques is always to be encouraged when trying to understand the range of motions present in a crystal lattice.

Solid-state NMR can be used to gain insights into crystallographic disorder occurring in bulk pharmaceuticals^21,37−44^ and can be combined with gauge-including projector-augmented-wave (GIPAW) calculations to verify and refine the crystal structure, that is, NMR crystallography.^19,24,37,45−70^ As a technique that observes the bulk sample, solid-state NMR can be used to validate the occurrence of crystallographic disorder and determine its nature.^21,25,71^ The disorder caused by dynamics can be investigated by solid-state NMR,^71−80^ offering qualitative information through line-shape analysis, chemical shift measurement,^21^ and dipolar coupling measurements.^61,81^ In addition, quantitative information on the thermodynamic parameters of the dynamics can be obtained through relaxation time measurements.^61,71,72,75^ There are limits to the ability of solid-state NMR to investigate disorder, which may arise due to the nature of the technique. Examples of this may arise from an insufficient resolution required to adequately resolve the crystallographic disorder, the disorder involving unreceptive nuclei or potentially missing the presence of dynamics occurring at a timescale distinct from that of the one-dimensional NMR experiment. Nevertheless, NMR crystallography has successfully been applied, for example, to understand the nature of the structural disorder in eniluracil,^82^ and quantitative information on the dynamics in pyrrolidine rings were obtained in a development compound using ^13^C spin–lattice time measurements.^20^ Recent computational advances involving molecular dynamics calculations have also been shown to be useful in interpreting experimental solid-state NMR lineshapes.^83,84^

**Figure 1:**
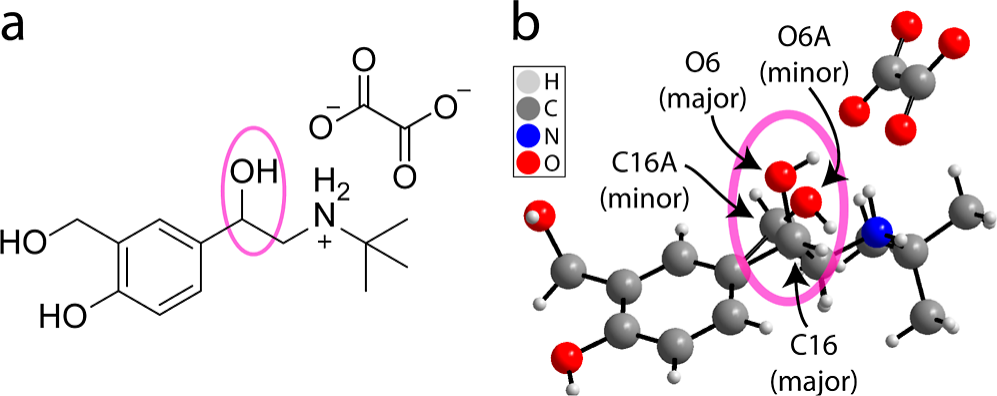
(a) Diagram of the molecular structure of salbutamol oxalate and (b) depictions of the X-ray crystal structure of salbutamol oxalate showing the crystallographic disorder. The magenta circle highlights the disordered site of interest, and the arrows indicate the site of the highest occupancy (“major phase”) and lowest occupancy (“minor phase”).

**Figure 2:**
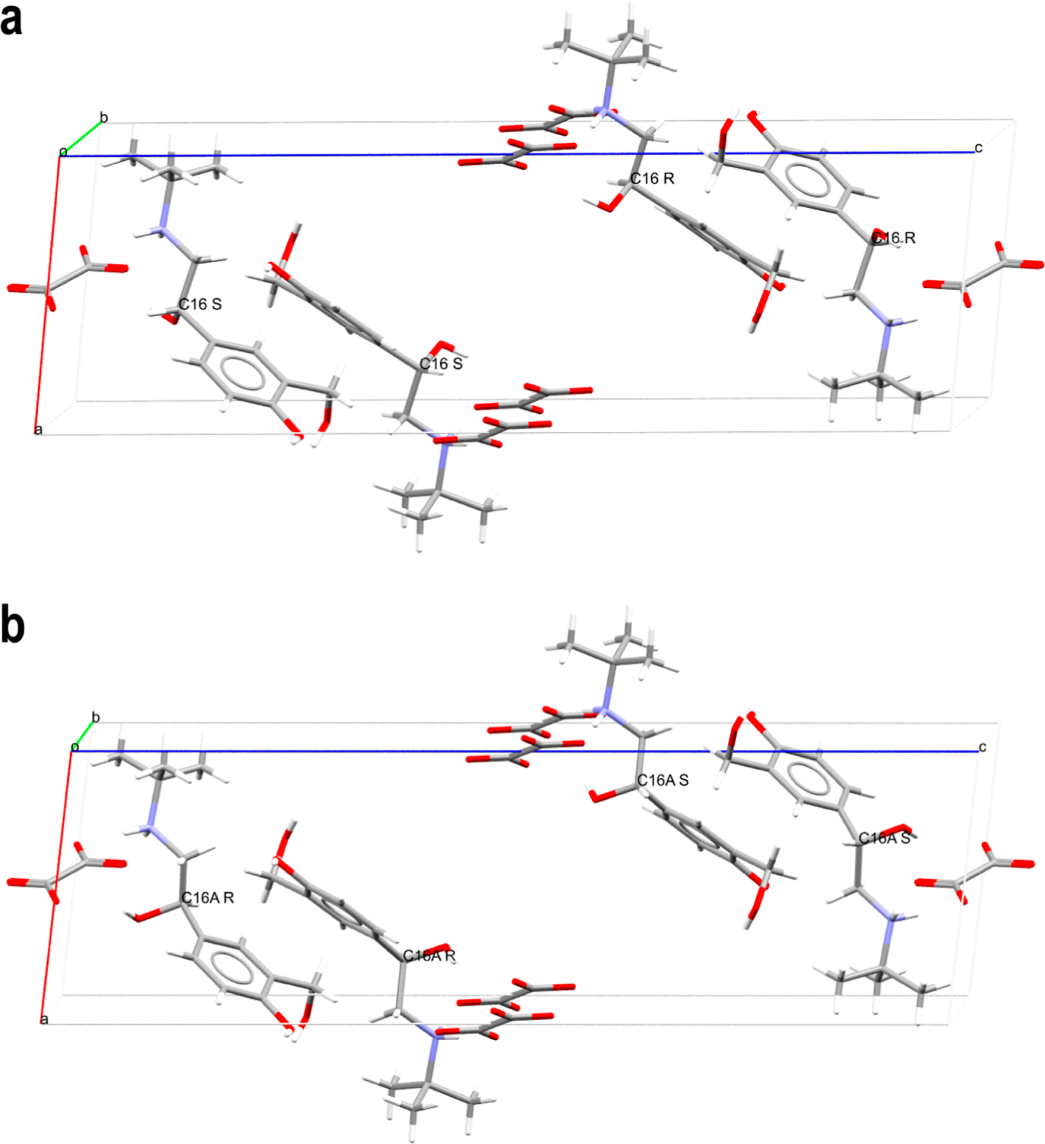
Packing of the structure displayed for the (a) highest occupancy “major phase” and (b) lowest occupancy “minor phase”. The crystallographic disorder is displayed. The impact of the disorder on the stereochemistry assignment is labeled on the chiral centers and both phases contain a racemic mixture of the two enantiomers of salbutamol, that is, the disorder is not due to one enantiomer being favored.

Salbutamol is a pharmaceutical product that is listed as an essential medicine by the World Health Organization and is used in part to treat respiratory distress, such as asthma.^85^ The main commercial form is salbutamol sulfate, although there are also structures of salbutamol reported in the Cambridge Structural Database^86^ featuring molecules of succinic acid,^87^ adipic acid,^87^ and even the pharmaceutical oxaprozin.^88^ Here, we report salbutamol oxalate as a model system and investigate this new crystalline form using a combination of X-ray crystallography and NMR crystallography with the aim of utilizing these analytical tools to gain a wider understanding of the nature of the disorder present in single crystals and in the bulk powder. The crystal structure of salbutamol oxalate features disorder at its *C–OH chiral center and here, a major and minor phase is seen, as shown in Figure 1. In this context, the term phase refers to the two distinguishable crystallographic positions observed in salbutamol oxalate. The disorder in salbutamol oxalate strongly resembles the disorder observed in the commercial form of salbutamol as salbutamol sulfate.^89^ It should be noted that while the disorder impacts the assignment of the stereochemistry on the chiral centers, the symmetry present ensures that all structures display a racemic mixture of the two enantiomers of salbutamol (see Figure 2). The impact of crystallization conditions on the degree of the disorder in the structure is investigated in order to gain insights into the distribution of conformational disorder present in multiple single crystals, which may be highly dependent on the crystallization conditions employed.

In this work, we establish the crystal structure of salbutamol oxalate and explore the variations of the crystallographic disorder between single crystals and the bulk powder using a combination of single crystal XRD and solid-state NMR. The site occupancies of the disorder in the *C–OH group were determined for multiple crystals in several samples, and the variability in the occupancies between single crystals is estimated. In order to assess the impact of crystallization conditions on the disorder, three batches of salbutamol oxalate were prepared under unique conditions, by varying either the crystallization temperature or the crystallization rate. Data were collected for at least three single crystals from each batch, and the disorder occupancies quantified in each single crystal (i.e., the major occupancy to minor occupancy ratio). The samples of salbutamol oxalate were further investigated by ^13^C solid-state NMR to quantify the disorder in the bulk sample. The identification and characterization of structural variations between single crystals in the bulk are of vital importance in understanding and predicting the properties of bulk powders. Structural variations could influence the chemical and physical properties of a material and its performance in the manufacturing process and the product. While single crystal XRD provides a crystal-to-crystal quantification of the disorder, solid-state NMR provides a view of the bulk material. In this work, we show that combining single crystal XRD with solid-state NMR provides an improved understanding of the crystallographic disorder between the local (i.e., as viewed by NMR) and longer-range periodic (i.e., as viewed by diffraction) scale.

## Experimental Section

### Sample Preparation

Salbutamol sulfate (>98%) was purchased from Tokyo Chemical Industry, and oxalic acid (>98%) was purchased from Sigma-Aldrich. Salbutamol was obtained from salbutamol sulfate in a solution of aqueous sodium carbonate (see Section S1 in the Supporting Information). It is expected that sodium sulfate remained in solution, while salbutamol precipitated. A very weak signal is observed in the ^23^Na solid-state NMR spectrum of **1a** (Figure S5 in Section S2) with a lineshape that is similar but not perfectly matching to that of sodium sulfate. Samples of salbutamol oxalate were prepared via evaporative crystallization at a 1:1 M ratio in 3 mL of water. A powder XRD pattern for each sample is available in Section S2 of the Supporting Information (Figures S4). The temperature at which samples were set to evaporate was between 20 and 50 °C, and the time for evaporation (a time point at which the solvent had visually evaporated) was varied between <1 and 3 days.

### X-ray Crystallography

A Rigaku Oxford Diffraction SuperNova was used for data collection, with measurements taken at 150 K or 298 K. The diffractometer was equipped with the Eos S2 detector and used a sealed tube source (Cu Kα radiation) with a graphite monochromator. Direct methods (SHELXS)^90^ were used for structure solution, and least squares minimization (SHELXL)^91^ was used for the structure refinement. Isotropic displacement parameters were initially used for the refinement of non-hydrogen atoms with multiple site occupancies. Subsequently, the ADPs were refined freely and fixed in position after several refinement cycles. The positions of hydrogen atoms were constrained in an idealized position and refined using a riding model. Crystal structure information for salbutamol oxalate: colorless block, C_14_H_22_N_1_O_5,_*M* = 284.32 g mol^–1^, monoclinic, space group *P*2_1_/n (#14), *a* = 8.4010 (3) Å, *b* = 6.2001 (2) Å, *c* = 27.6519 (9) Å, β = 97.211(3)^o^, *V* = 1428.90 (8) Å^3^, *Z* = 4, *Z*′ = 1, ρ_calc_ = 1.323 g cm^3^, F(000) = 613.0, μ = 0.832 mm^–1^.

### ^13^C Solid-State NMR

All samples were packed into 4 mm zirconium oxide magic-angle spinning (MAS) rotors. Experiments were performed on either a Bruker Avance III spectrometer operating at a ^1^H Larmor frequency of 500 MHz using the 4 mm Bruker HXY probe, or on a Bruker NEO spectrometer operating at a ^1^H Larmor frequency of 850 MHz using a 4 mm Bruker HXY probe in double-resonance mode. A MAS rate of 12.5 kHz was used throughout all the experiments. At 11.7 T, 1D ^13^C cross-polarization MAS (CPMAS) spectra were acquired using a ramped contact pulse from 50 to 100% on the ^1^H channel,^92^ a contact time of 2 ms, a ^1^H π/2 pulse duration of 2.5 μs, a 3 s recycle delay, co-adding 1024 transients, and using SPINAL64 proton decoupling^93^ with a ^1^H nutation frequency of 100 kHz and a pulse duration of 3.8 μs. A ^1^H–^13^C HETCOR experiment was performed using the same CP parameters but with a 200 μs contact time. The ^13^C *T*_1_ relaxation times were measured using the same cross-polarization (CP) parameters followed by an inversion recovery pulse sequence, with a ^13^C π/2 pulse of 4.2 μs, sampling 8 datapoints along the *T*_1_ curve. For the variable-temperature experiments at 11.7 T, 512 transients were acquired. At 20.0 T, the 1D ^13^C CPMAS spectra were acquired using a ramped contact pulse from 70 to 100% on the ^1^H channel,^92^ a contact time of 4 ms, a ^1^H π/2 pulse duration of 3 μs, a 3 s recycle delay, co-adding 512 transients, and using SPINAL64 proton decoupling^93^ with a ^1^H nutation frequency of 83 kHz and a pulse duration of 5.6 μs. The ^13^C spectra were calibrated using l-alanine and referenced to 178.8 ppm, relative to TMS at 0 ppm.^94,95^ The temperatures were calibrated externally using the ^79^Br resonance of KBr.^96^ All relative intensities were estimated using the integral of the peak.

### ^1^H Solid-State NMR

^1^H solid-state NMR spectra were acquired on a Bruker Avance II+ spectrometer operating at a ^1^H Larmor frequency of 600 MHz using a 1.3 mm Bruker HXY probe or on a Bruker NEO spectrometer operating at a ^1^H Larmor frequency of 850 MHz using a 4 mm Bruker HXY probe in double-resonance mode. A π/2 pulse of 2.5 μs and a recycle delay of 3 s was used. 32 (600 MHz) or 4 (850 MHz) transients were co-added. The ^1^H spectra were referenced using the CH_3_ resonance of l-alanine to 1.1 ppm, relative to adamantane at 1.85 ppm.^97^

### NMR Calculations

All DFT^98−100^ calculations were performed using the GIPAW^101^ method as implemented in CASTEP^102^ as part of Materials Studio version 17^103^ and following recommended procedures.^60,104^ The crystal structures obtained from the experimental X-ray crystallography results were used as the structural models for the calculations. The GGA PBE functional^105^ was employed for all calculations, beginning with a geometry optimization prior to calculating the NMR chemical shifts. The geometry optimization was performed with TS DFT-D correction,^106^ on-the-fly ultrasoft pseudopotentials, Koelling–Harmon relativistic treatment, and a constrained unit cell, and all atomic positions were optimized. The cutoff energy was 600 eV and the *k*-point separation was 0.05 Å^–1^. NMR calculations were subsequently performed using the same parameters as the geometry optimization but with a cutoff energy of 700 eV. The calculated σ_iso_ values were extracted using the script Magres2Topspin^60^ and converted into δ_iso_ using the equation δ_iso_ = σ_ref_ – σ_calc_, with a σ_ref_(^13^C) of 170.6 ppm and a σ_ref_(^1^H) of 30.2 ppm. Calculating the σ_ref_ specific to each system is a common practice in the literature,^107^ noting that the average σ_ref_ values are also available.^108^

## Results and Discussion

### General X-ray Structural Characterization

**Figure 3:**
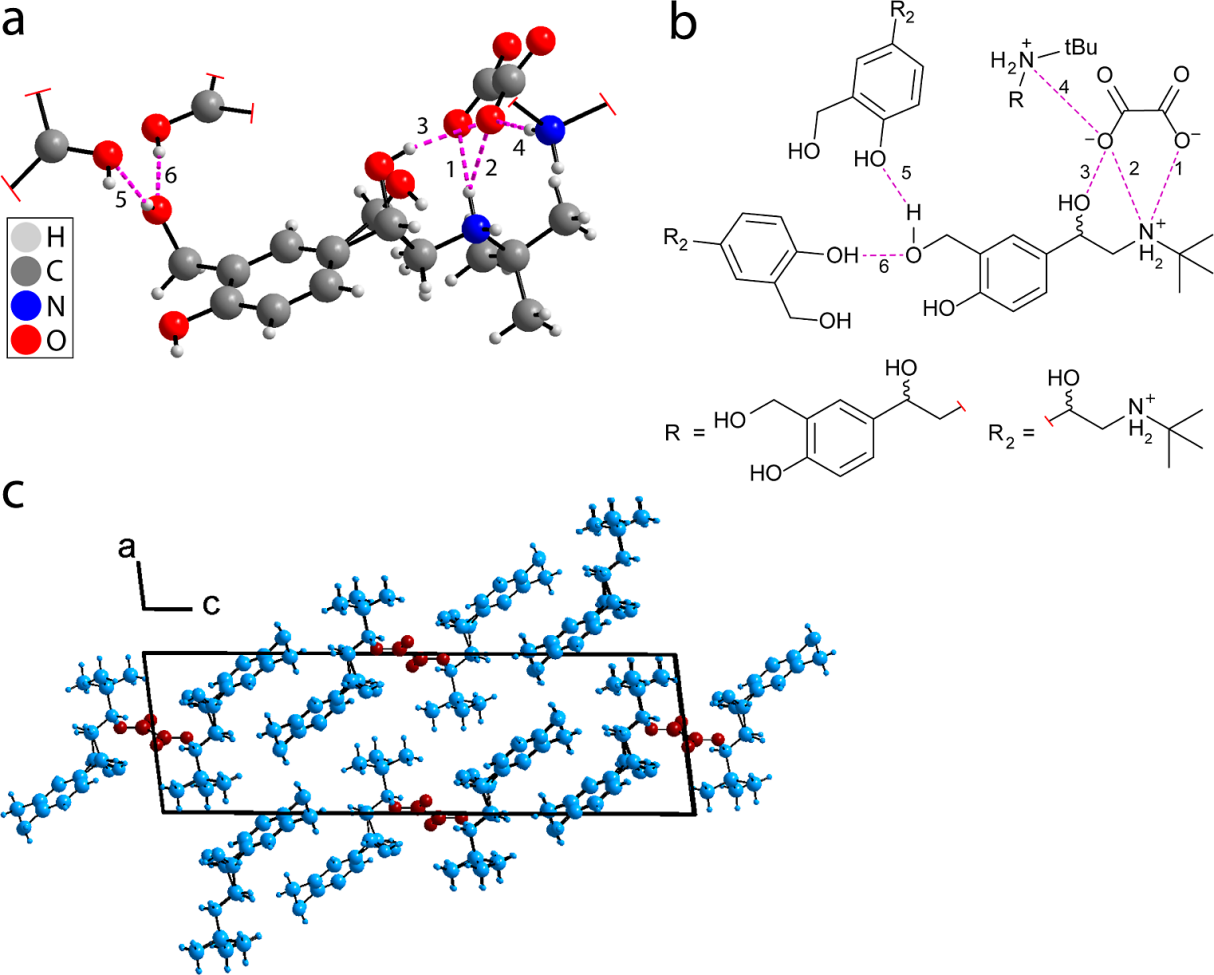
(a) Depiction of the crystal structure of salbutamol oxalate (sample **1a**, trial **#1**, at 150 K, see Table S10, CSD deposition number 2106949) showing the hydrogen bond framework and (b) a schematic of the hydrogen-bonding framework. The hydrogen-bonding interactions are labeled as 1 to 6 (see Table S2 for hydrogen bond lengths and angles) (c) Crystallographic packing viewed along the *b* axis. The salbutamol cation is shown in light blue, and the oxalate anion is shown in dark red.

As shown in Figure 3a, the asymmetric unit of the crystal structure of salbutamol oxalate features a salbutamol molecule and half an oxalate anion, with the X-ray-determined parameters reported in Table 1 (and Tables S10, S11, and S12 in Section S6 of the Supporting Information). The protons of oxalic acid have been transferred to the NH_2_^+^ group of salbutamol, which is supported by their p*K*a difference, DFT calculations, and the ^13^C solid-state NMR chemical shifts (vide infra). The crystal structure of salbutamol oxalate features crystallographic disorder of the *C–OH group over two positions, labeled in Figure 1b as C16–O6 (major) and C16A-O6A (minor), and the relative occupancies of the minor phase at 150 K are given in Table 2. Note that all the considered crystal structures contain a racemic mixture of the two enantiomers of salbutamol due to the symmetry constraints of the crystal; the disorder is not due to one enantiomer being favored. Several different ways of refining the crystallographic disorder were tested, and the most sensible model was chosen (see discussion in Section S3 of the Supporting Information).

**Table 1:** Crystal Data and Structure Refinement for a Variable-Temperature X-ray Diffraction Study with Salbutamol Oxalate, Sample **1b**, Trial **#2** at 298 and 150 K

parameter	**1b** at 298 K	**1b** at 150 K
empirical formula	C_14_H_22_NO_5_	C_14_H_22_NO_5_
temperature (K)	298.09 (15)	150.01 (10)
crystal system	monoclinic	monoclinic
space group	*P*2_1_/n	*P*2_1_/n
*a* (Å)	8.4844 (8)	8.4010 (3)
*b* (Å)	6.2472 (6)	6.2001 (2)
*c* (Å)	27.619 (2)	27.6519 (9)
α (deg)	90	90
β (deg)	96.832(9)	97.211(3)
γ (deg)	90	90
volume (Å)	1453.5 (2)	1428.90 (8)
*Z*	4	4
ρ_calc_ (g/cm^3^)	1.299	1.323
μ (mm^–1^)	0.816	0.832
F(000)	612.0	613.0
radiation	Cu Kα (1.54184 Å)	Cu Kα (1.54184 Å)
2θ range for data collection (deg)	10.612 to 147.718	10.7 to 147.512
reflections collected	6899	7518
goodness-of-fit on F^2^	1.121	1.046
*R*_all data_	*R*_1_ = 0.0950, *WR*_2_ = 0.2585	*R*_1_ = 0.0532, *WR*_2_ = 0.1192
*R* [*I* ≥ 2θ(I)]	*R*_1_ = 0.0677, *WR*_2_ = 0.2073	*R*_1_ = 0.0459, *WR*_2_ = 0.1131
largest diff. peak/hole (e Å^–3^)	0.52/–0.53	0.30/–0.23
chemical occupancy (*C–OH)^a^	0.134:0.866(8)	0.140:0.860(5)
CSD deposition no.	2106958	2106953

a The standard uncertainties were noted down before site occupancies were fixed.

**Table 2:** Disorder Occupancy of the Minor Site C16A Observed in Single Crystals and Powdered Samples of Salbutamol Oxalate Crystallized under Different Conditions, as Determined by X-ray Crystallography and ^13^C Solid-State NMR

			X-ray disorder occupancy at 150 K					
	conditions		individual single crystal trials^a^					
sample	time (days)	temp (°C)	**#1**	**#2**	**#3**	average (%)	distribution (%)^b^	NMR occupancy at 300 K (%)^c^
**1a**	<1	50	0.161 (6)	0.121 (5)	0.132 (6)	13.8	(−) 1.7	12 ± 3
							(+) 2.3	
**1b**	≈3	20	0.150 (4)	0.140 (5)	0.129 (5)	13.9	(−) 1.0	9 ± 3
							(+) 1.1	
**1c**	≈3	30	0.153 (5)	0.148 (4)	0.165 (6)	15.5	(−) 0.7	11 ± 2
							(+) 1.0	

a Standard uncertainties were noted down before site occupancies were fixed.

b Distribution from the average value.

c Occupancies determined by ^13^C CPMAS solid-state NMR by measuring the relative intensity of the resonance, C16, corresponding to the minor phase at 11.7 T at room temperature (see Figure 4).

The hydrogen bonding framework between salbutamol and the oxalate anion consists of six hydrogen bonds (see Figure 3b), three of which are charge-assisted and occur between the charged NH_2_^+^ group and the oxalate ion. Interestingly, hydrogen bonds 2 and 3 in Figure 3b are bifurcated hydrogen bonds with a three-centered interaction.^109^ One of these bifurcated bonds, hydrogen bond 3, resides with the disordered *C–OH region. Variable temperature XRD studies show a variation in the observed characteristics of the bond length and bond angle of hydrogen bond 3 (see Tables S2 and S3 in Section S3 of the Supporting Information). This may provide an explanation for the molecular motions observed in the crystal structure of salbutamol oxalate; the weak intermolecular interactions observed may favor a locally loose crystal packing in the disordered region, leading to an increase in molecular motions.^82^Figure 3c shows the packing arrangement, with salbutamol (light blue) assembling around channels of oxalate anions (dark red).

The disorder observed in the crystal structure of salbutamol oxalate is similar to that observed in other solid forms of salbutamol, namely, salbutamol sulfate,^89^ salbutamol sulfate monohydrate,^85^ and salbutamol benzoate.^110^ In these cases, the *C–OH group in salbutamol is also disordered and shows multiple phases. The hydrogen bond framework in salbutamol sulfate^89^ is similar to that shown here in salbutamol oxalate, with two hydrogen bond acceptors (a charge-assisted NH_2_^+^ and a hydroxyl group) competing for the same hydrogen-bond donor atom (see Section S4 in the Supporting Information).

### Disorder Characterization by X-ray Crystallography

Single crystal XRD studies have been carried out on three individual single crystals (referred to as trial **#1**, **#2,** and **#3**), from each of three different sample batches, henceforth referred to as samples **1a**, **1b,** and **1c**, prepared using the following crystallization conditions: sample **1a**, fast evaporation (hours) of an aqueous solution at 50 °C; sample **1b**, slow evaporation (3 days) of an aqueous solution at 20 °C; sample **1c**, slow evaporation (3 days) of an aqueous solution at 30 °C. Experiments with various crystallization temperatures were carried out to explore how the site occupancy factors of salbutamol oxalate vary in crystals as a function of the temperature and rate of crystallization. Table 2 summarizes the crystallization conditions of each sample and the resultant disorder occupancy of the single crystals analyzed under the refinement model discussed in Section S3 in the Supporting Information. The refined average disorder and disorder distribution from the data collected is calculated.

An average minor phase of approximately 14–15% was found in samples **1a**, **1b,** and **1c** from the analysis of several single crystals. Small occupancy distributions are observed for the samples, with individual refined occupancies showing a maximum variation of 4% for sample **1a**, 2% for sample **1b,** and 2% for sample **1c**. The standard uncertainties on these values also indicate consistently precise determination for these samples. The conditions at which crystallization occurred did not seem to have a significant influence on the disorder occupancies measured for each sample. The effect of crystallization conditions on site occupancies has been investigated for other disordered molecules in the literature. A similar observation was found in the case of eniluracil;^82^ no significant differences were observed between the site occupancies when crystallized under different procedures. In contrast, in the case of 5-chlorouracil,^111^ significant differences were observed between site occupancies, which seemed to depend on the crystallization procedure. Different crystallization solvents and methods were investigated in both of these cases. Further investigations are required to determine why changing the crystallization procedure appears to affect the site occupancies of some disordered molecules but not others. Certainly, single crystal XRD is not a bulk analysis technique, and the analysis of three single crystals per sample is not a complete representation of the bulk material. The methodology in this study does, however, provide a valuable insight into the nature of the disordered material. One aim of the present study is to investigate whether the combination of solid-state NMR with X-ray crystallography can help to bridge the gap between characterizing the disorder in single crystals and the bulk material. Solid-state NMR experiments were, therefore, performed to evaluate the nature of the disorder (i.e., static or dynamic) and to quantify the phase occupancies in the bulk material.

### ^13^C Solid-State NMR Spectroscopy

^13^C CPMAS solid-state NMR experiments have been applied to investigate the crystallographic disorder observed in the X-ray crystal structure of salbutamol oxalate and have been performed at magnetic fields of 11.7 and 20.0 T. Three samples of salbutamol oxalate were analyzed as above, **1a**, **1b,** and **1c**, with their ^13^C NMR spectra shown in Figure 4. Note that, for such an experiment, ∼100 mg of the powdered sample is packed into a MAS rotor of outer diameter, 4 mm. Overall, there are 12 unique ^13^C resonances observed in the experimental spectra, and their assignments are shown in Figure 4 with their chemical shifts reported in Section S5, Table S4 in the Supporting Information. Of the 12 resonances in the ^13^C spectrum, the C15 (oxalate), C3 (aromatic C–OH), C16 (*CHOH), and C12/C13/C14 (*tert*-butyl methyl) resonances feature a shoulder near the base of resonances, while for resonances C2 (aromatic C), C4 (aromatic C–H), C9 (CH_2_), and C11 (*tert*-butyl), a second resonance of lower intensity was resolved. These shoulders and lower-intensity resonances have been assigned to the minor position of the crystallographic disorder. Using ^13^C NMR resonance C9, the disorder has been quantified in each sample at room temperature, assuming a consistent CP transfer between them, as summarized in Table 2. As shown in Table S9 and Figure S19 of the Supporting Information, the ^1^H *T*_1ρ_ values were measured for both phases in the cases where the ^13^C resonances were resolved. The ^1^H *T*_1ρ_ values are comparable between the two phases, supporting similar CP transfer kinetics for both phases. The recycle delay was optimized, with no differences observed in the relative intensity for either component in the ^13^C NMR spectrum when acquired with a recycle delay of 3 s or 5 s. The amount of disorder measured by solid-state NMR appears to be consistent across all three samples and is in close agreement with the X-ray crystallography data, as shown in Table 2. The XRD data for all single crystals analyzed for salbutamol oxalate are presented in Section S6 of the Supporting Information. These results highlight the importance of pairing single crystal XRD with a bulk analysis technique, such as solid-state NMR. While single crystal XRD can provide a general picture of the positions of the atoms and molecules in the solid state, solid-state NMR has provided information on disorder occupancies in the bulk powder.

**Figure 4:**
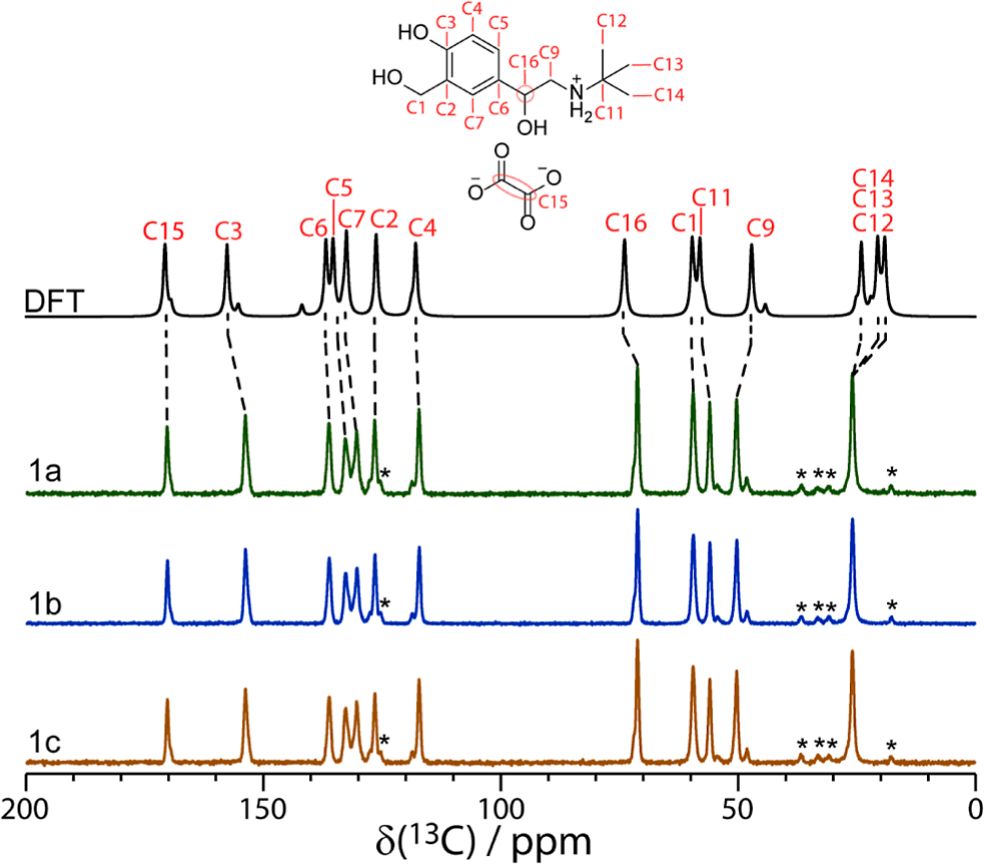
Experimental ^13^C CPMAS (contact time = 2 ms, ν_MAS_ = 12.5 kHz) solid-state NMR spectra of sample **1a**, **1b**, and **1c,** obtained at 11.7 T. The GIPAW DFT-calculated spectrum is shown above with the intensity of the minor phase set to 15% that of the major phase. The asterisks denote spinning sidebands, and the assignments are shown above.

The experimental ^13^C chemical shifts are in excellent agreement (*R*^2^ > 0.99, see Figure S17 of the Supporting Information) with the DFT-calculated chemical shifts,^22,46,112−114^ as shown in black in Figure 4, and supports the current structural model and the *Z*′ of 1. The experimental ^13^C NMR spectrum was assigned using the ^1^H–^13^C HETCOR experiment (see Figure S12 of the Supporting Information) and GIPAW DFT calculations. The DFT spectrum was simulated by splitting the disorder into two separate structural models, one containing the major site of occupancy and the other containing the minor site of occupancy, and calculating the ^13^C chemical shifts (see Table S4 in the Supporting Information for the DFT-calculated ^13^C chemical shifts). The NMR spectrum was simulated from the calculated chemical shifts using the solid lineshape analysis (SOLA),^115^ and the relative intensity of the ^13^C spectrum for the minor position was set to 15% of that of the major position. Overall, the DFT calculations are in excellent agreement with the experiments, and the presence of shoulders and splitting arising due to the disorder is reproduced well by the DFT calculations.

Additional DFT calculations were performed to investigate the variability of the ^13^C chemical shift as a function of the N–H proton distance, moving from the salt form (^+^N–H···O^–^) to the cocrystal form (N···H–O). As shown in Figure S13 of the Supporting Information, the GIPAW-calculated ^13^C chemical shift of the oxalate carbon (resonance C15) is very sensitive to the proton position and varies from 170.3 ppm in the salt form to 160.2 ppm in the cocrystal form. The experimental ^13^C chemical shift of the oxalate carbon (resonance C15) is consistent with the DFT calculations obtained on the salt form and is also supported by a prior report on oxalic acid cocrystals.^116^ The DFT calculations were repeated on structures **1b** acquired at 298 K and 150 K, and only very small ^13^C chemical shift changes on an order of 0.1 ppm (corresponding to the accuracy of reporting experimental ^13^C solid-state NMR chemical shifts) were observed, as shown in Figures S14 and S15 of the Supporting Information.

**Figure 5:**
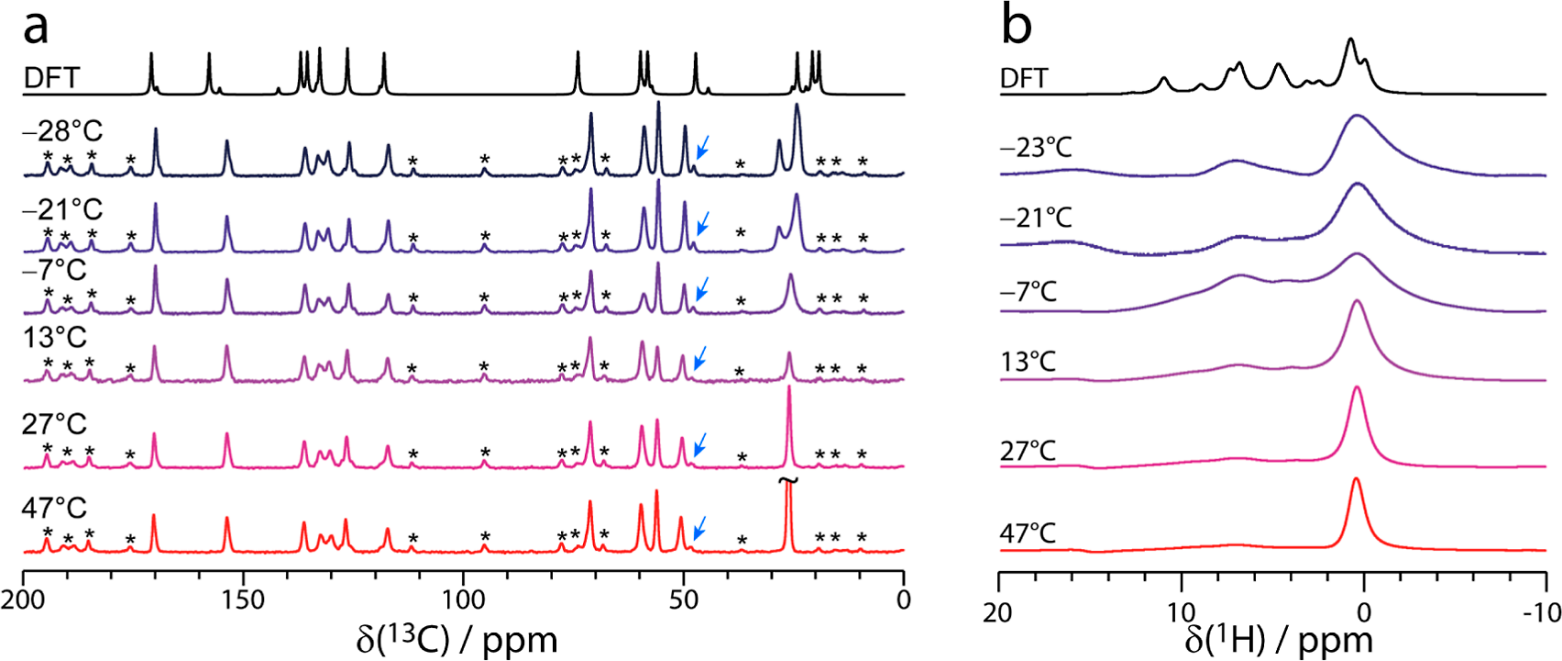
Experimental solid-state MAS (ν_MAS_ = 12.5 kHz) NMR spectra of **1a** acquired at 20 T at several temperatures observing (a) ^13^C using ^1^H–^13^C CP (contact time = 4 ms) and (b) ^1^H using a spin-echo (with a spin-echo duration of 2 μs) pulse sequence. The asterisks in (a) denote the spinning sidebands, and the blue arrows highlight the increase in intensity for the minor position of the CH_2_ carbon (C9), adjacent to the disordered *C–OH chiral center. Simulated spectra corresponding to the GIPAW-calculated chemical shifts are shown above in black in both (a,b).

One difference between the experimental and calculated ^13^C spectrum is the methyl carbon on the *tert*-butyl group (resonance C12/C13/C14 in Figure 4), which has three peaks in the calculated data but only a single peak in the experimental spectrum. This difference arises due to the calculations being performed at 0 K and not taking into consideration the dynamics of the *tert*-butyl group occurring at room temperature. Upon reducing the sample temperature to −28 °C, the methyl ^13^C resonance splits into two with 1:2 relative intensity, as shown in Figure 5a, and the agreement between the experimental ^13^C NMR results and the DFT calculations improves significantly. At higher temperatures, the methyl resonances coalesce as a result of the rapid rotation of the *tert*-butyl group, relative to the NMR timescale;^23,117−121^ this is also observed in ^1^H solid-state NMR spectra (see Figure S10 and variable-temperature ^1^H MAS NMR spectra recorded at 12.5 kHz MAS in Figures 5b and S11 of the Supporting Information). In addition, ^13^C *T*_1_ spin–lattice relaxation times [*T*_1_(^13^C)] were measured as a function of temperature and show a strong dependence of *T*_1_(^13^C) on the temperature for resonances C11 and C12/C13/C14, further supporting the occurrence of a dynamic *tert*-butyl group (see Figure S18 of the Supporting Information). Notably, the *tert*-butyl dynamics is not readily distinguishable from the X-ray data (see thermal ellipsoid plots in Figure S20 of the Supporting Information).

As shown in Figure 5a, the relative intensity of all the ^13^C resonances assigned to the minor position slightly increases as the temperature is reduced. This is most noticeable for ^13^C resonance C9 at δ(^13^C) = 50.2 ppm (see blue arrows in Figure 5a), with the integrated intensity of the minor phase, relative to the major phase, increasing from 0.14 at 47 °C to 0.20 at −28 °C in **1a**. Additional variable-temperature experiments were performed at both 20.0 and 11.7 T, as presented in Figures S8 and S9 of the Supporting Information, respectively. The changes in the relative intensities indicate that the population of the major and minor phases is dependent on the temperature. This dependence on the temperature is not observed in the X-ray crystallography results of a single crystal from sample **1b** (see Table 1), rather the refined disorder site occupancies are within the error at the two temperatures and appear to be independent of the acquisition temperature within the precision of the crystallographic determination. This apparent discrepancy in the temperature dependence of the disorder may be due to the constraints that were imposed during the X-ray data refinement, in part masking the nature of the disorder and removing unusual features to meet CIF requirements.

The increase in the relative intensity of the resonances assigned to the minor phase as the temperature decreases may be attributed to the disorder being in part dynamic and could be in the slow-exchange regime at fields of 11.7 and 20.0 T. Unfortunately, the *T*_1_(^13^C) relaxation times related to C16 and C9 ^13^C resonances were lengthy, as shown in Figure S18 of the Supporting Information, and no clear dependence of *T*_1_(^13^C) with the temperature was observed. In order to be in the slow-exchange regime, the exchange rate must be lower than the ^13^C Larmor frequencies of 125 MHz and 213 MHz, respectively. This is in direct contrast to the coalescence of the ^13^C resonance of the *tert*-butyl group, which is in the fast-exchange regime at temperatures above 0 °C at 20.0 T. Between temperatures of 0 and −14 °C at 20.0 T, the chemical exchange is in the intermediate regime, and broadening is observed for the ^13^C resonance at 26 ppm, as shown in Figure 5a, whereas at temperatures lower than −21 °C at 20.0 T, the *tert*-butyl group motion slows, and multiple resonances are observed. The results on the *tert*-butyl dynamics are in agreement with prior reports on different systems.^23,117−121^ As shown in Figure 5b, corresponding narrowing of the *tert*-butyl ^1^H resonance (at ∼0 ppm) is observed above 0 °C.

## Conclusions

The crystal structure and nature of the disorder in salbutamol oxalate were investigated by single crystal XRD and ^13^C solid-state MAS NMR spectroscopy. While the single crystal X-ray data highlight the presence of a crystal-to-crystal variation in the occupancies of 12–16%, ^13^C solid-state NMR quantifies the average occupancy of the minor position in the bulk powder at 11 ± 3% at room temperature. Both the XRD and solid-state NMR results are in close agreement and suggest that the nature of the disorder in salbutamol oxalate may, in part, be dynamic. Further, ^13^C and also ^1^H solid-state MAS NMR revealed the occurrence of rapid rotation of the *tert*-butyl group. Overall, this work illustrates the utility of combining X-ray crystallography and solid-state NMR to investigate the crystallographic disorder at the molecular scale and larger scale in bulk powders. A criticism of our work could be that the multi-batch single-crystal XRD analysis was carried out at 150 K, while the solid-state NMR analysis was carried out at room temperature. An even more extended study than that presented here would have been to carry out a multi-batch single-crystal XRD analysis at a range of temperatures so that the role of temperature could be better understood. However, we do note that the occupancies for the major and minor phases are within the error for the one single crystal for which single-crystal diffraction analysis was performed at 150 and 298 K. Moreover, GIPAW-calculated chemical shifts for DFT geometry-optimized crystal structures at these two temperatures are within 0.1 ppm.

## Supporting Information Available

Experimental synthesis procedure of salbutamol, additional NMR spectra, additional single-crystal XRD data, powder XRD data, and thermal ellipsoid plots (PDF)
