# Supplementary material for: Combining X-ray and NMR Crystallography to Explore the Crystallographic Disorder in Salbutamol Oxalate
Source: Cryst Growth Des. 2022 Jul 20;22(8):4696–707. doi: 10.1021/acs.cgd.1c01093 (PMC9374327; doi:10.1021/acs.cgd.1c01093)
Supplement: Supplementary file 1 — cg1c01093_si_001.pdf [file cg1c01093_si_001.pdf]

Combining X-ray and NMR Crystallography to Explore the Crystallographic Disorder in Salbutamol Oxalate

Supporting Information

Aneesa J. Al-Ani,[1] Patrick M.J. Szell[2], Zainab Rehman,[2] Helen Blade[3], Helen P. Wheatcroft[3], Leslie P. Hughes[3*], Steven P. Brown[2*], Chick C. Wilson[1*]

*Author to whom correspondence may be addressed

[1] Centre for Sustainable and Circular Technologies (CSCT), University of Bath, Claverton Down, Bath BA2 7AY, United Kingdom. E-mail: C.C.Wilson@bath.ac.uk
[2] Department of Physics, University of Warwick, Coventry, UK, CV4 7AL. E-mail: S.P.Brown@warwick.ac.uk
[3] Oral Product Development, Pharmaceutical Technology & Development, Operations, AstraZeneca, Macclesfield, UK. E-mail: Les.Hughes2@astrazeneca.com

Table of Contents

Section S1. Salbutamol synthesis S3

Section S2. Experimental and calculated PXRD traces and ^{23}Na MAS NMR of salbutamol oxalate. S5

Section S3. Additional information on the crystal structure of salbutamol oxalate S7

Section S4. The hydrogen bond framework in salbutamol sulfate S10

Section S5. Additional solid-state NMR results S11

Section S6. X-ray diffraction data for all single crystals analysed of salbutamol oxalate S22

Section S1. Salbutamol synthesis

The base in salbutamol sulfate (Tokyo Chemical Industry, >98.0%) was liberated from a saturated solution of aqueous sodium carbonate (Fluka, >99.5%) to afford salbutamol. Figure S1 shows a schematic of the reaction. The resultant product was characterised by PXRD (Figure S2) and DSC (Figure S3). It is expected that sodium sulfate remained in solution whilst salbutamol, which is poorly soluble in water, precipitated out of solution. A very weak signal is observed in a ^{23}Na solid-state NMR spectrum of **1a** (Figure S5 in section S2) with a lineshape that is similar but not perfectly matching to that of sodium sulfate.

$C_{13}H_{21}NO_3$: ^{1}H NMR (DMSO-d6, 300 MHz): δ 0.99 (9H), 4.44 (3H), 5.00 (2H), 6.67 (1H), 6.96 (1H), 7.24 (1H). ^{13}C NMR (D2O-d2, 300 MHz): δ 25.92 (3C), 47.94 (1C), 55.12 (1C), 60.69 (1C), 71.04 (1C), 118.48 (1C), 121.68 (1C), 126.86 (1C), 127.35 (1C), 128.72 (1C), 162.44 (1C).

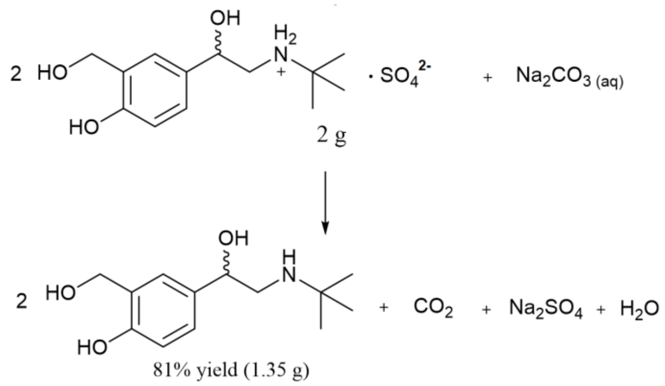

Figure S1. A schematic of the chemical reaction used to form salbutamol.

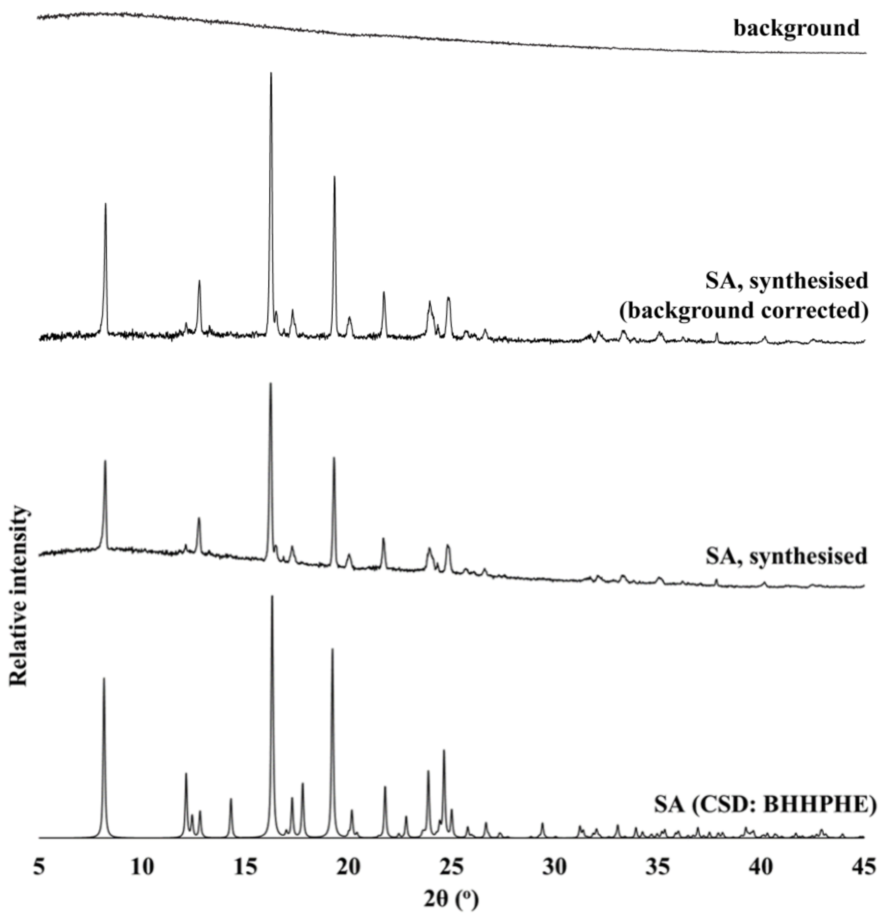

Figure S2. A PXRD trace of synthesised salbutamol is compared to a calculated trace from the CSD. The instrumental background (top) and a PXRD trace with background subtraction are shown.

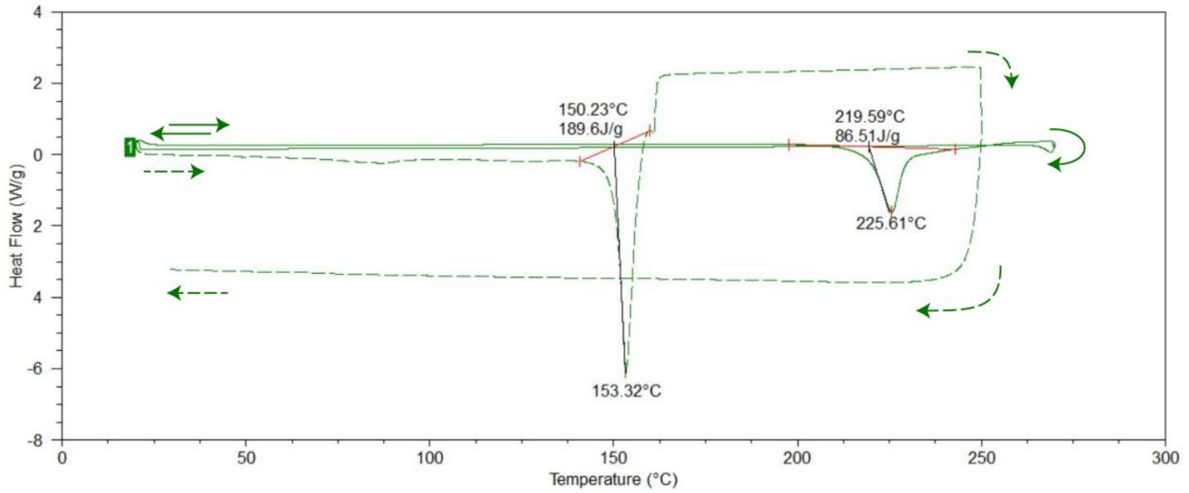

Figure S3. The DSC trace of synthesised salbutamol (dashed trace, 143°C onset) and salbutamol oxalate (solid trace, 183°C onset) upon performing a heating and cooling cycle. The literature reports a melting point range of 150-157°C for salbutamol and 180°C for salbutamol sulfate (ChemSpider, RSC). The arrows denote the direction of the curve, increasing in temperature during the heating cycle and decreasing in temperature during the cooling cycle.

Section S2. Experimental and calculated PXRD traces and ^{23}Na MAS NMR of salbutamol oxalate.

Experimental Powder X-Ray Diffraction Procedure. All PXRD data were collected with a STOE STADI P diffractometer equipped with monochromatic Cu-Kα1 radiation (λ = 1.54045 ˚A). All samples were analysed at 298 K by transmission PXRD.

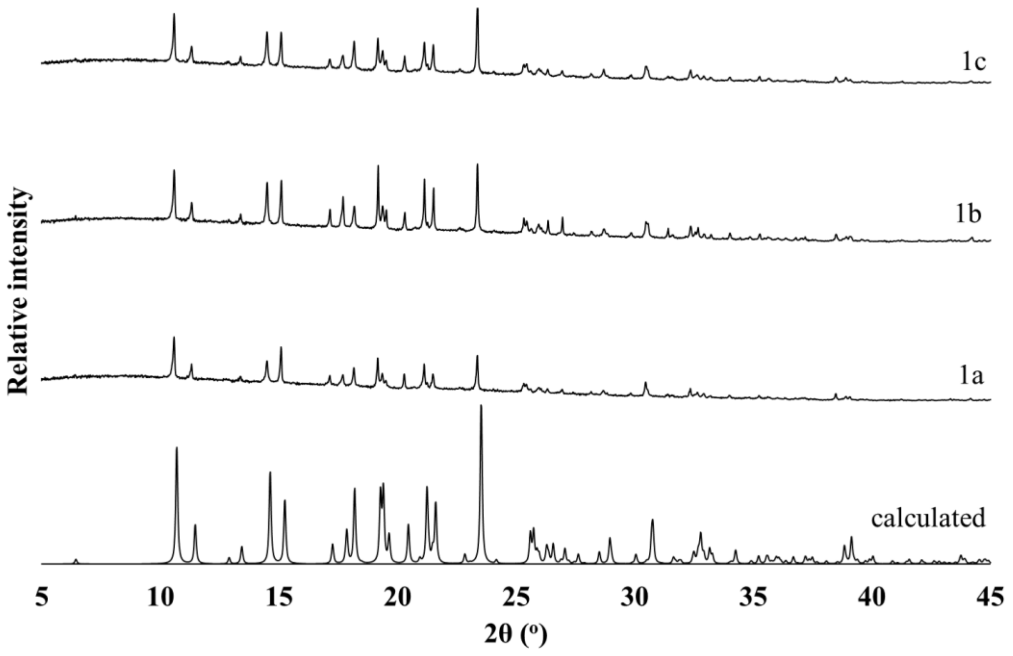

Figure S4. Experimental PXRD traces for **1a**, **1b** and **1c** and the calculated trace from single crystal XRD studies that correspond to salbutamol oxalate. Note the baseline due to the instrument background (see Figure S2).

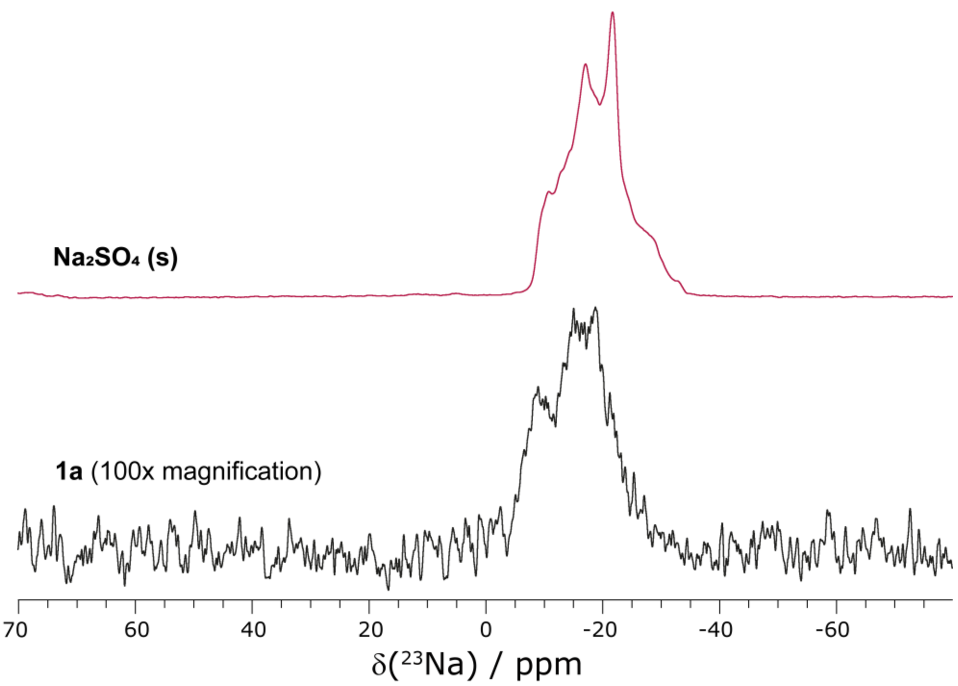

Figure S5. One-pulse ^{23}Na MAS (12.5 kHz) NMR spectra of Na_2SO_4 (red, above) and **1a** (black, below) obtained at 11.7 T using a 4 mm HXY probe. 1024 transients were acquired for **1a**, whereas 4 transients were acquired for Na_2SO_4. The central transition $\pi/2$ pulse length was 3 μs (for the central transition) for sample **1a**. A recycle delay of 20 s was used.

Section S3. Additional information on the crystal structure of salbutamol oxalate

Structure refinement models

Three different ways of refining the crystallographic disorder in salbutamol oxalate were tested (see below). No significant differences were seen between the models that would suggest one model was preferable over another.

Model 1 (CIF file = 1a #1 i): The occupancy values for C16 and C16A were fixed to follow the occupancy values for O6 and O6A (see Table S1).

Model 2 (CIF file = 1a #1 ii): The occupancy values for O6 and O6A were fixed to follow the occupancy values for C16 and C16A (see Table S1).

Model 3 (CIF file = 1a #1 iii): All atoms with multiple occupancies were refined freely and no restraints were placed to fix the occupancy positions.

Model 1 was chosen to refine all crystal structures in this work. Model 3 was not chosen since significantly different site occupancy values were seen for the carbon and oxygen atoms which seemed unlikely. Model 1 was chosen over model 2 since the oxygen atoms held a larger amount of electron density than the carbon atoms. The structure refinement parameters for each model are shown in Table S1.

Table S1. The refinement parameters for models 1, 2 and 3 with salbutamol oxalate, sample **1a,** trial **#1** at 150 K.

Model[a]	1	2	3
Goodness-of-fit on F^2	1.021	1.026	1.030
$R_{\text{all data}}$	R_1= 0.0561<br>WR_2= 0.1234	R_1= 0.0560<br>WR_2= 0.1228	R_1= 0.0560<br>WR_2= 0.1230
R [I>=2θ(I)]	R_1= 0.0469<br>WR_2= 0.1166	R_1= 0.0469<br>WR_2= 0.1160	R_1= 0.0469<br>WR_2= 0.1163
Largest diff. peak/hole (e Å^{-3})	0.35 / –0.31	0.35/ –0.32	0.35/ –0.31
Chemical occupancy (*C-OH)	0.161 : 0.839	0.2033 : 0.7967	O-H = 0.161 : 0.839(6)<br>C-H = 0.203 : 0.797(13)

[a] CSD deposition Numbers: 2106949, 2106951 and 2106954.

Variable temperature X-ray studies

The same single crystal from sample **1b** (trial #2) was characterised by single crystal XRD at 150 K and 298 K. Table S2 and S3 lists the hydrogen bond distances and angles at 150 K and 298 K. Figure S6 shows the labels for atoms and hydrogen bonds that are referred to in Tables S2 and S3.

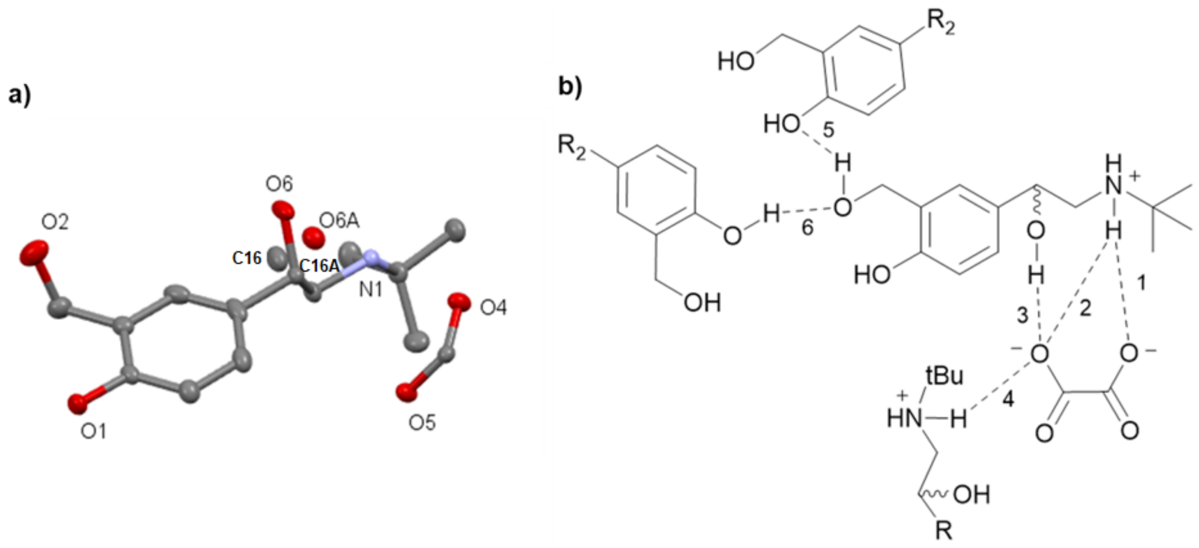

Figure S6. Diagrams of salbutamol oxalate showing (a) labelled atoms and (b) labelled hydrogen bonds in the structure.

Table S2. The hydrogen bond distances and angles observed in the crystal structure of salbutamol oxalate sample **1b** at 150 K.

hydrogen bond #	atom labels X-H···Y[a]	bond length $d_{X\text{-}H\cdots Y}$ / Å	bond length $d_{X\cdots Y}$ / Å	bond angle $\theta_{X\text{-}H\cdots Y}$ / °
1	N1-H···O4	1.90(2)	2.816(2)	168(2)
2	N1-H···O5	2.42(2)	2.948(2)	116(2)
3	O6-H···O5	1.97(3)	2.837(2)	175(3)
4[b]	N1’-H···O5	1.95(2)	2.821(2)	170(2)
5[b]	O2-H···O1’	2.02(3)	2.867(2)	157(3)
6[b]	O1-H···O2’	1.78(3)	2.624(2)	173(3)

[a] The labels “X” and “Y” signify the hydrogen bond donor and acceptor, respectively, either N or O.
[b] The (’) denotes the atom of an adjacent molecule of salbutamol.

Table S3. The hydrogen bond distances and angles observed in the crystal structure of salbutamol oxalate sample **1b** at 298 K.

hydrogen bond #	atom labels X-H···Y[a]	bond length $d_{X\text{-}H\cdots Y}$ / Å	bond length $d_{X\cdots Y}$ / Å	bond angle $\theta_{X\text{-}H\cdots Y}$ / °
1	N1-H···O4	2.03(4)	2.845(3)	164(3)
2	N1-H···O5	2.39(3)	2.942(3)	123(3)
3	O6-H···O5[c]	2.114	2.895(4)	159.1
4[b]	N1'-H···O5	1.88(3)	2.819(3)	169(3)
5[b]	O2-H···O1'	2.10(6)	2.905(4)	141(5)
6[b]	O1-H···O2'	1.80(5)	2.649(4)	167(5)

[a] The labels "X" and "Y" signify the hydrogen bond donor and acceptor, respectively, either N or O.
[b] The (') denotes the atom of an adjacent molecule of salbutamol.
[c] The hydrogen atom is fixed in an idealised position based on a riding model.

Section S4. The hydrogen bond framework in salbutamol sulfate

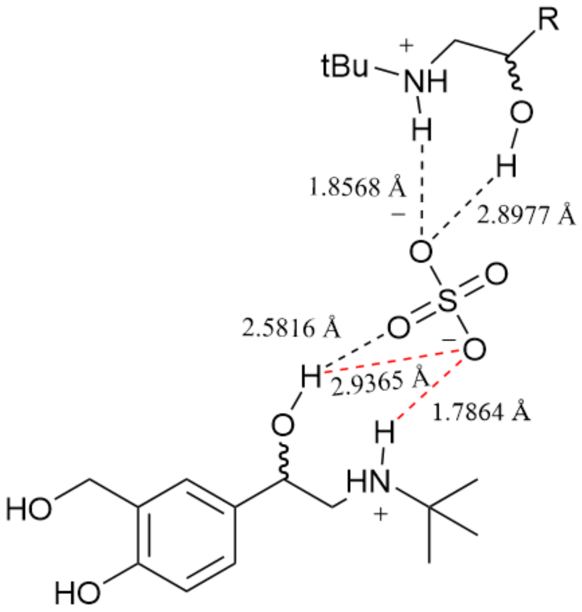

Figure S7. The hydrogen bond frame work in salbutamol sulfate (CSD: SALBUT).[1] The competing hydrogen bonds that reside between a disordered C-OH region are highlighted in red. No bond errors are available in the published data for the hydrogen bond distances, $d_{H\cdots O}$. Note that all the considered crystal structures contain a racemic mixture of the two enantiomers of salbutamol, i.e., the disorder is not due to one enantiomer being favoured.

Section S5. Additional solid-state NMR results

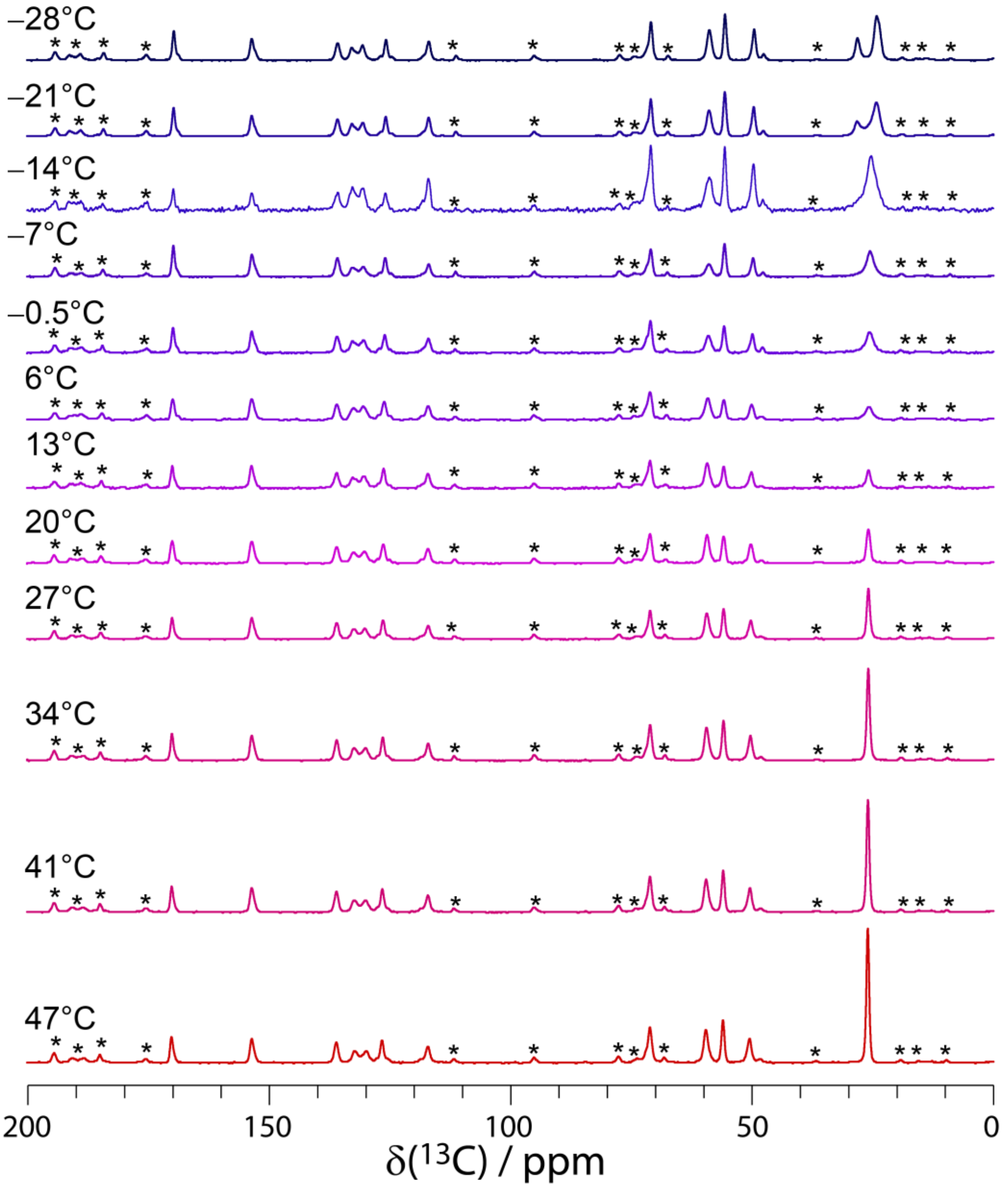

Figure S8. Experimental ^{13}C CPMAS solid-state NMR spectra of sample **1a** obtained at 20 T under cross-polarization magic-angle spinning conditions (contact time = 4 ms, ν_{MAS} = 12.5 kHz) acquired at multiple temperatures. The asterisks denote spinning sidebands that originate from the magic-angle spinning.

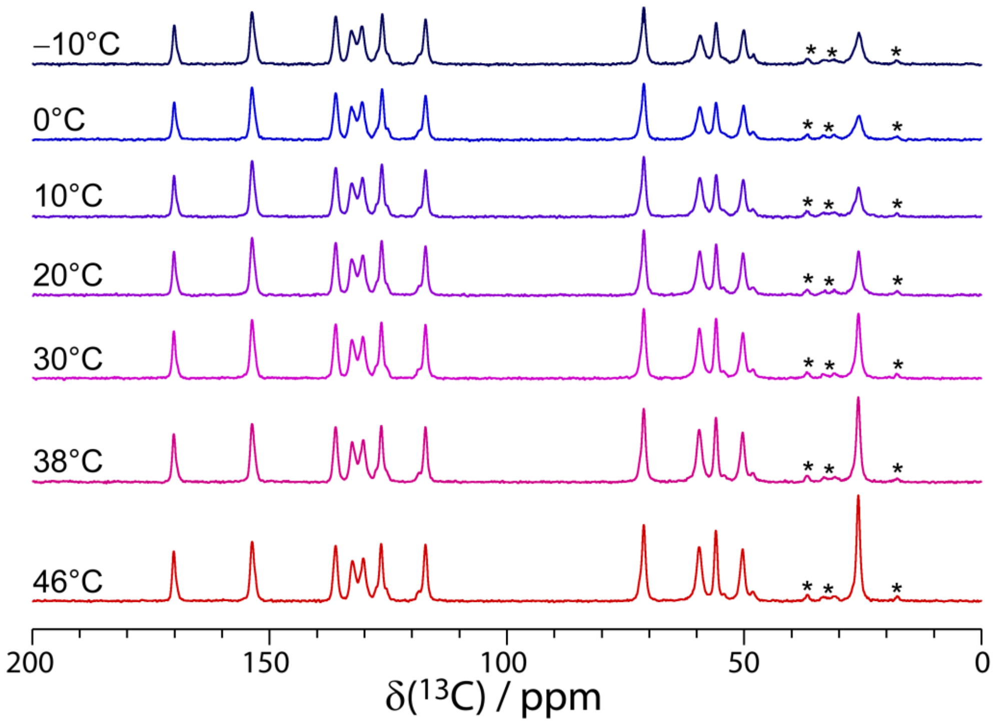

Figure S9. Experimental ^{13}C CPMAS solid-state NMR spectra of sample **1a** obtained at 11.7 T under cross-polarization magic-angle spinning conditions (contact time = 2 ms, ν_{MAS} = 12.5 kHz) acquired at multiple temperatures. The asterisks denote spinning sidebands that originate from the magic-angle spinning.

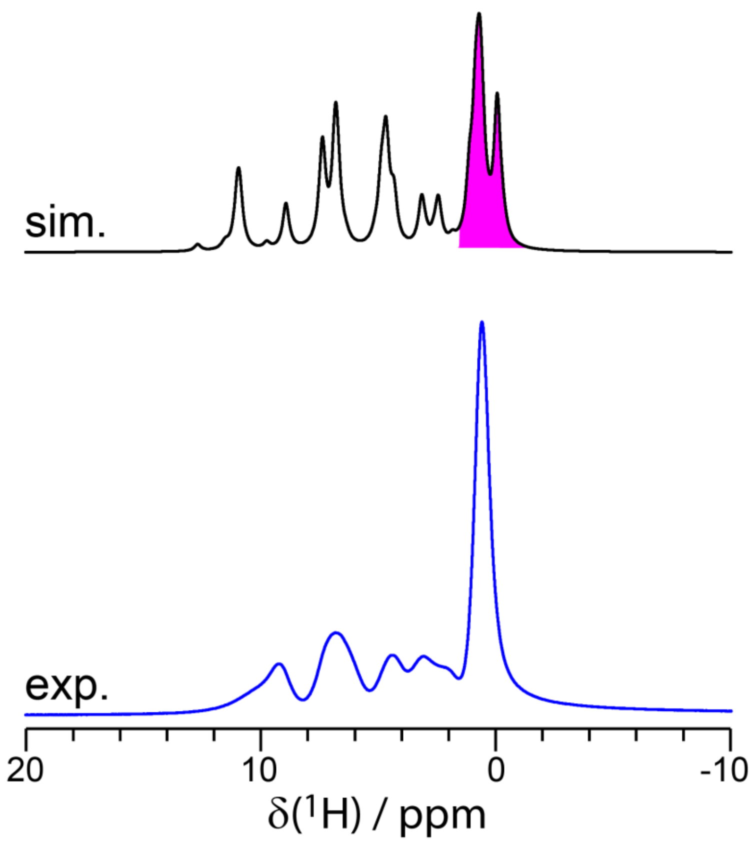

Figure S10. An experimental ^{1}H one-pulse solid-state MAS NMR spectrum of salbutamol oxalate (**1a**, ν_L = 600 MHz, ν_{MAS} = 60 kHz, blue trace), showing the GIPAW DFT-calculated spectrum above (σ_{ref}(^{1}H) = 30.2 ppm, black trace). The resonances assigned to the *tert*-butyl group are highlighted in magenta on the DFT-calculated spectrum.

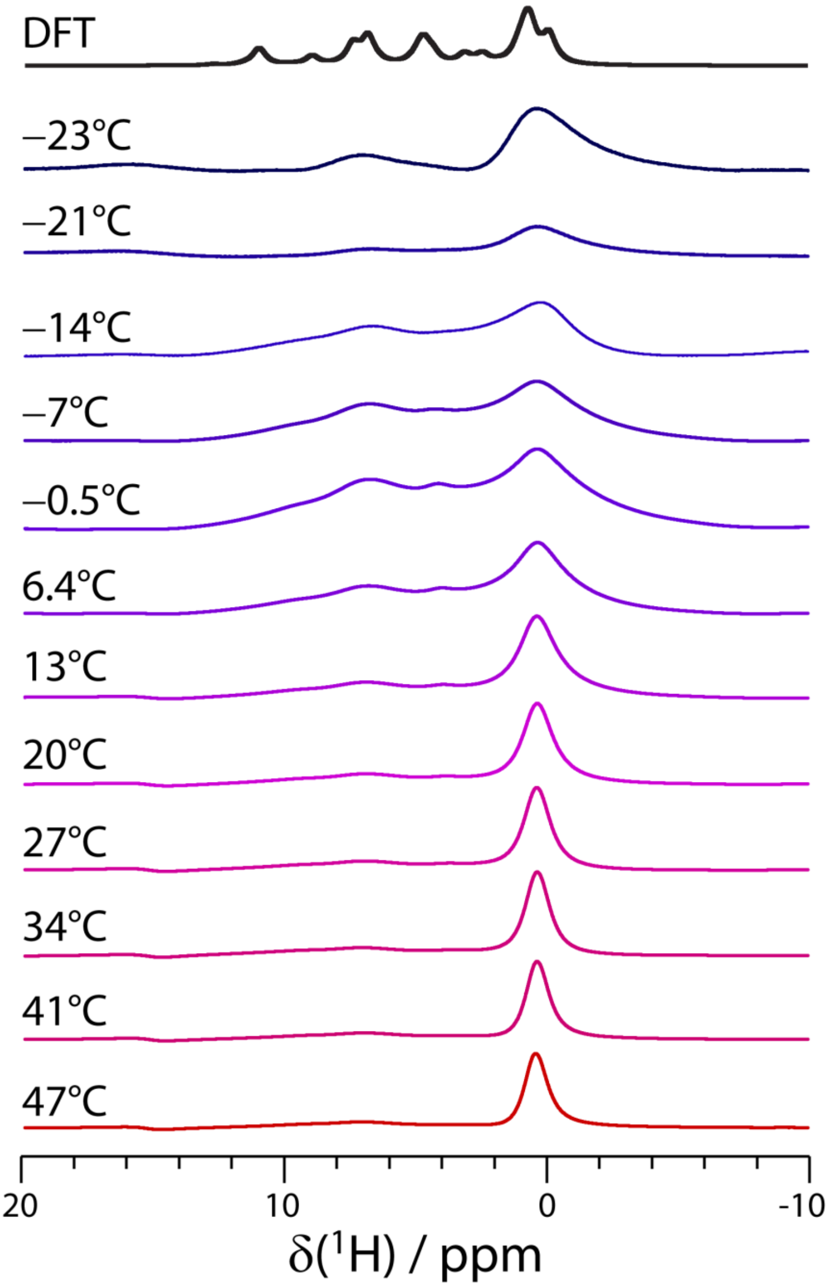

Figure S11. Experimental ^{1}H spin-echo solid-state MAS NMR spectra of salbutamol oxalate (**1a**, ν_L = 850 MHz, ν_{MAS} = 12.5 kHz) acquired at multiple temperatures, showing the GIPAW DFT-calculated spectrum above ($\sigma_{ref}(^{1}H)$ = 30.2 ppm, black trace).

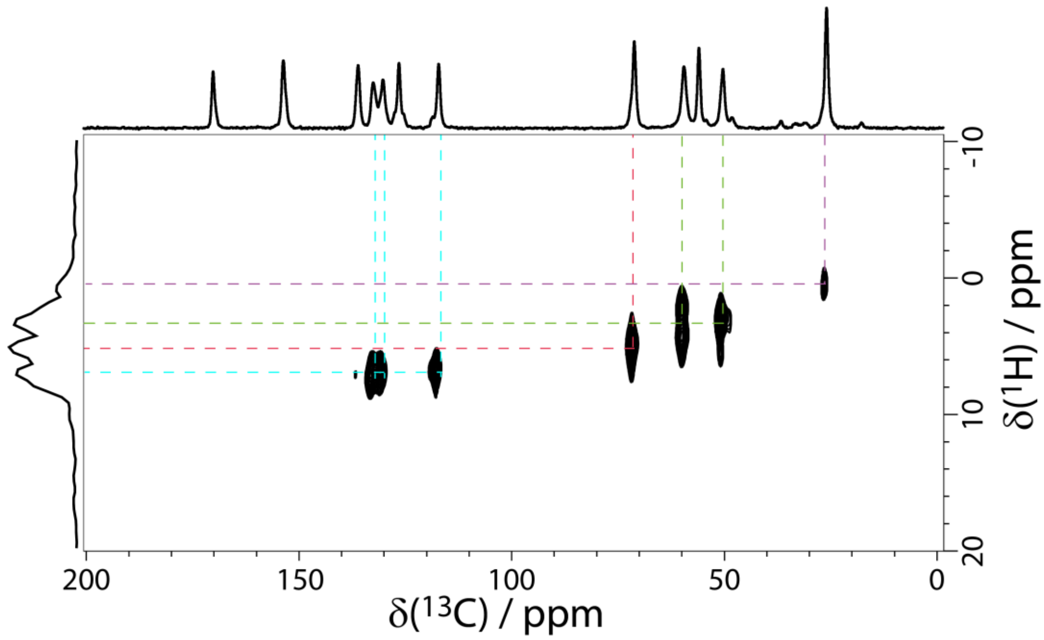

Figure S12. A ^1H-^{13}C CP-HETCOR with ^1H FSLG decoupling solid-state MAS NMR spectrum of salbutamol oxalate (**1a**, contact time = 200 μs, ν_L = 500 MHz, ν_{MAS} = 12.5 kHz,). The dashed lines are added as a guide. The x-axis displays an external ^{13}C projection using the experimental ^{13}C CPMAS spectrum, while the y-axis is a skyline projection. 128 transients were co-added for each of 64 t_1FIDs for a recycle delay of 3 s, corresponding to an experimental time of 6.8 h. The States-TPPI method was used to achieve sign discrimination in F_1.

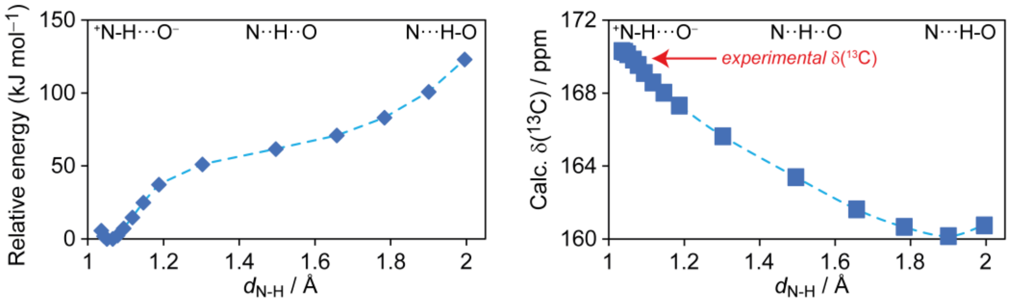

Figure S13. DFT-calculated (a) relative energy and (b) δ(^{13}C) chemical shift (GIPAW) of the oxalate carbon as a function of the N-H bond length. The salt form is shown on the left ($^+$N-H···O$^-$), whereas the cocrystal form is shown on the right (N···H-O). The experimental δ(^{13}C) is highlighted by the red arrow in (b).

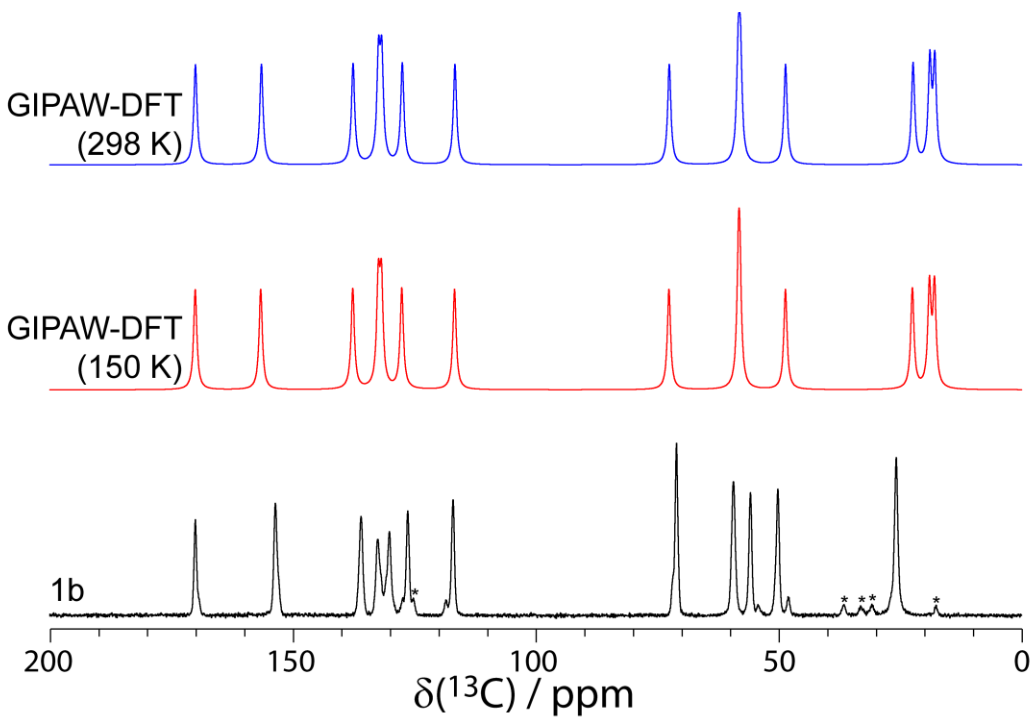

Figure S14. Experimental ^{13}C cross-polarization magic-angle spinning solid-state NMR spectrum of **1b** acquired at room temperature (black trace, 11.7 T, $\nu(^{1}H)$ = 500 MHz, contact time = 2 ms, ν_{MAS} = 12.5 kHz), and GIPAW-DFT calculated ^{13}C solid-state NMR spectrum of **1b** performed on the geometry-optimized crystal structure of **1b** acquired at 150 K (red trace) and 298 K (blue trace).

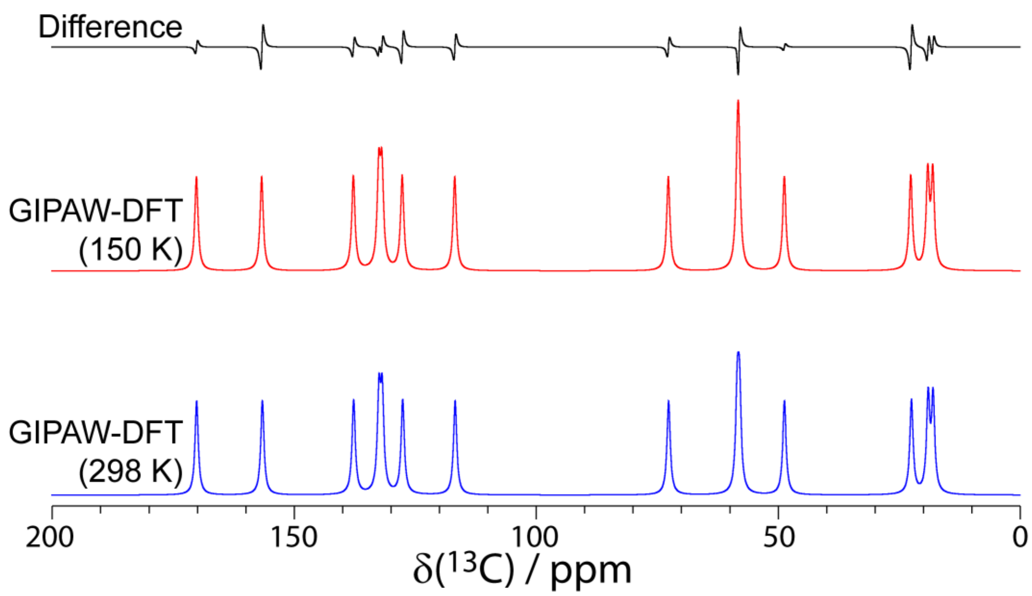

Figure S15. GIPAW-DFT calculated ^{13}C solid-state NMR spectrum of **1b** performed on the geometry-optimized crystal structure of **1b** acquired at 150 K (red trace) and 298 K (blue trace). The difference spectrum is shown above in black.

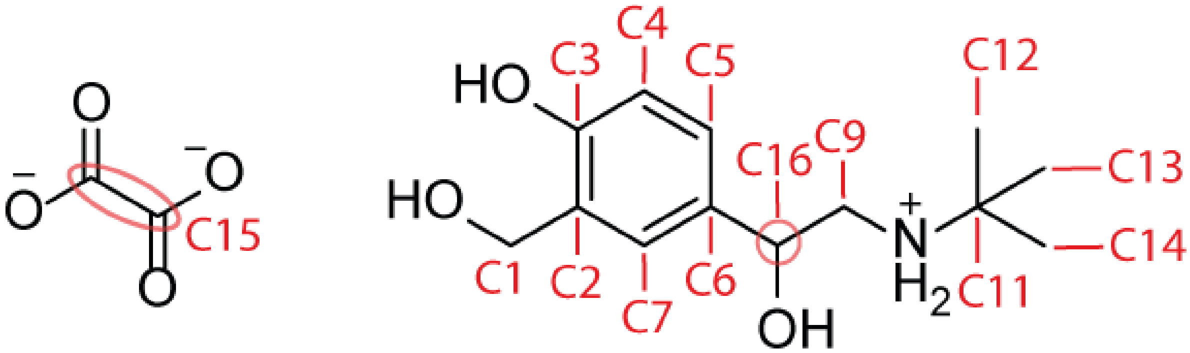

Figure S16. Molecular structure of salbutamol oxalate with the numbers corresponding to the ^{13}C assignments and also the atom labels in the X-ray diffraction structures.

Table S4. Experimental and GIPAW DFT-calculated ^{13}C solid-state NMR chemical shifts (in ppm) of salbutamol oxalate, including the coefficient of determination (R^2) and root mean square error (RMSE).

assignment	experimental $\delta(^{13}C)$ / ppm[a] (major positions)	calculated $\delta(^{13}C)$ / ppm (major positions)[b]	experimental $\delta(^{13}C)$ / ppm[a] (minor positions)	calculated $\delta(^{13}C)$ / ppm (minor positions)[b]
C15	170.1	170.7	169.5	169.5
C3	153.6	157.6	153.0	155.3
C6	136.0	136.8	136.0	141.9
C5	132.5	135.3	132.5	133.3
C7	130.2	132.5	130.3	132.4
C2	126.4	126.2	127.4	126.4
C4	117.1	117.9	118.5	118.9
C16	71.1	73.9	71.9	74.4
C1	59.4	59.7	59.4	59.2
C11	55.9	58.1	54.4	57.2
C9	50.2	47.2	48.1	44.4
C12, C13, C14	25.9	24.1<br>20.7<br>19.2	26.8	25.3<br>22.2<br>20.0
	R^2	0.996[c]		0.996[c]
	RMSE	3.0[d]		3.2[d]

[a] The reported experimental ^{13}C chemical shifts were measured at 293 K at 11.7 T on sample **1a** (see Figure S9).

[b] A chemical shielding reference, $\sigma_{ref}(^{13}C)$, of 170.6 ppm was used to convert the calculated chemical shieldings into chemical shifts.

[c] See Figure S17 for the line of best fit and the determination of R^2.

[d] The RMSE was calculated as in Salager et al[2]:

$$RMSE_{\mathrm{NMR}} = \sqrt{\frac{1}{N}\sum_i^N\left(\delta_{calc,i} - \delta_{\mathrm{exp,i}}\right)^2}$$

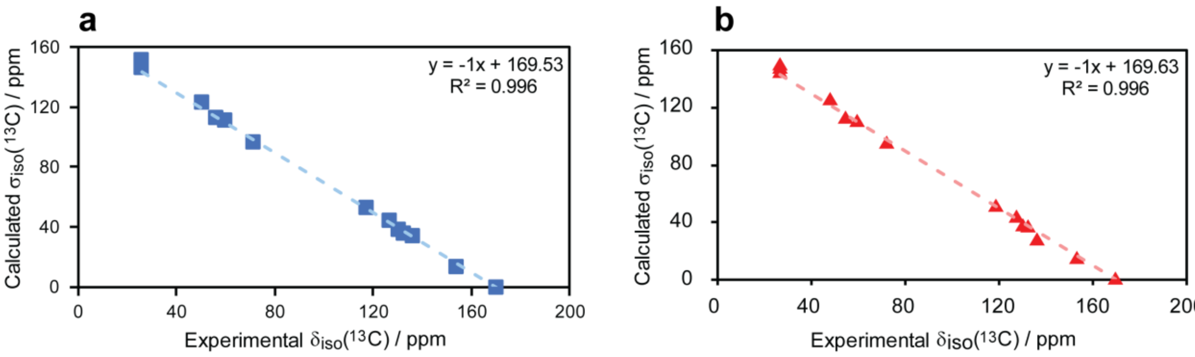

Figure S17. Plots of calculated $\sigma_{iso}(^{13}C)$ chemical shielding against experimental $\delta_{iso}(^{13}C)$ chemical shift for the (a) major and (b) minor positions (see Table S4). The linear regressions were performed with the gradient fixed to unity.

Table S5. Relative intensities of the ^{13}C signals in sample **1a** assigned to the minor phase as a function of temperature for resonances C2 ($\delta(^{13}C)$ = 127.4 ppm), C4 ($\delta(^{13}C)$ = 118.5 ppm), C9 ($\delta(^{13}C)$ = 48.1 ppm) and C11 ($\delta(^{13}C)$ = 54.4 ppm), obtained at 11.7 T (see Figure S9).

Temperature (°C)	Resonance C2	Resonance C4	Resonance C9	Resonance C11
46	0.14	0.12	0.17	0.10
38	0.17	0.13	0.18	0.13
30	0.19[a]	0.16	0.18	0.13
20	0.17[a]	0.13	0.19	0.14
10	0.14[a]	0.18	0.20	0.13[a]
0	0.14[a]	0.18[a]	0.22	0.16[a]
–10	0.13[a]	0.17[a]	0.25	0.16[a]

[a] Resonance is poorly resolved and is associated with a higher degree of uncertainty.

Table S6. Linewidths (in ppm) at half maximum of ^{13}C resonances C15 ($\delta(^{13}C)$ = 170.1 ppm), C16 ($\delta(^{13}C)$ = 71.1 ppm), C9 ($\delta(^{13}C)$ = 50.2 ppm), C11 ($\delta(^{13}C)$ = 55.9 ppm), and C12/C13/C14 ($\delta(^{13}C)$ = 25.9 ppm) in sample **1a** as a function of temperature obtained at 11.7 T (see Figure S9).

Temp. (°C)	Resonance C15	Resonance C16	Resonance C9	Resonance C11	Resonance C12 / C13 / C14
46	0.82	0.97	1.09	0.82	0.96
38	0.82	0.96	1.10	0.86	0.92
30	0.81	0.96	1.16	0.87	1.04
20	0.83	0.97	1.10	0.94	1.13
10	0.85	1.01	1.12	0.99	1.39
0	0.88	1.07	1.19	1.03	1.69
–10	0.86	1.12	1.21	1.01	1.76

Table S7. Relative intensities of the ^{13}C signals assigned to the minor phase as a function of temperature for resonances C2 ($\delta(^{13}C)$ = 126.9 ppm), C4 ($\delta(^{13}C)$ = 118.5 ppm), and C9 ($\delta(^{13}C)$ = 47.6 ppm) obtained at 20.0 T (see Fig. S8) on sample **1a**. The minor phase for resonance C11 was unresolved.

Temperature (°C)	126.9 ppm Resonance C2	118.5 ppm Resonance C4	47.6 ppm Resonance C9
47	0.15	0.19	0.15
41	0.18	0.15	0.14
34	0.17	0.16	0.14
27	0.17	0.20	0.14
20	0.15	0.17	0.13
13[b]	0.23[b]	0.23[b]	0.12[b]
6[b]	0.20[b]	0.26[b]	0.21[b]
–0.5[b]	0.20[b]	0.17[b]	0.22[b]
–7[b]	0.20[b]	0.27[b]	0.20[b]
–14[b]	0.22[b]	0.22[b]	0.19[b]
–21	0.17	0.18	0.19
–28	0.17	0.17	0.20

[a] Resonance is poorly resolved and is associated with a higher degree of uncertainty.
[b] The spectrum at this temperature had a lower signal-to-noise ratio relative to the other spectra.

Table S8. Linewidths (in ppm) at half maximum of ^{13}C resonances C15 ($\delta(^{13}C)$ = 170.1 ppm), C16 ($\delta(^{13}C)$ = 71.2 ppm), C9 ($\delta(^{13}C)$ = 49.8 ppm), C11 ($\delta(^{13}C)$ = 55.8 ppm), and C12/C13/C14 ($\delta(^{13}C)$ = 26.0 ppm) as a function of temperature obtained at 20.0 T (see Figure S8) on sample **1a**.

Temp. (°C)	Resonance C15	Resonance C16	Resonance C11	Resonance C9	Resonance C12 / C13 / C14
47	0.80	0.98	0.75	1.06	0.68
41	0.78	0.98	0.75	0.98	0.70
34	0.72	0.98	0.83	1.07	0.70
27	0.76	0.95	0.80	1.05	0.79
20	0.91	1.15	0.95	1.07	0.95
13[b]	0.83[b]	1.15[b]	0.88[b]	1.02[b]	1.02[b]
6[b]	0.93[b]	1.29[b]	0.95[b]	1.08[b]	1.59[b]
–0.5[b]	0.76[b]	1.24[b]	0.76[b]	0.87[b]	1.84[b]
–7[b]	0.70[b]	1.00[b]	0.79[b]	0.84[b]	1.86[b]
–14[b]	0.64[b]	1.15[b]	0.79[b]	1.03[b]	2.44[b]
–21	0.70	0.90	0.81	0.86	1.94 (24.2 ppm)[a]<br>0.95 (28.2 ppm)[a]
–28	0.72	0.88	0.75	0.88	1.52 (24.2 ppm)[a]<br>1.01 (28.2 ppm)[a]

[a] Two resonances observed at low temperature.
[b] The spectrum at this temperature had a lower signal-to-noise ratio relative to the other spectra.

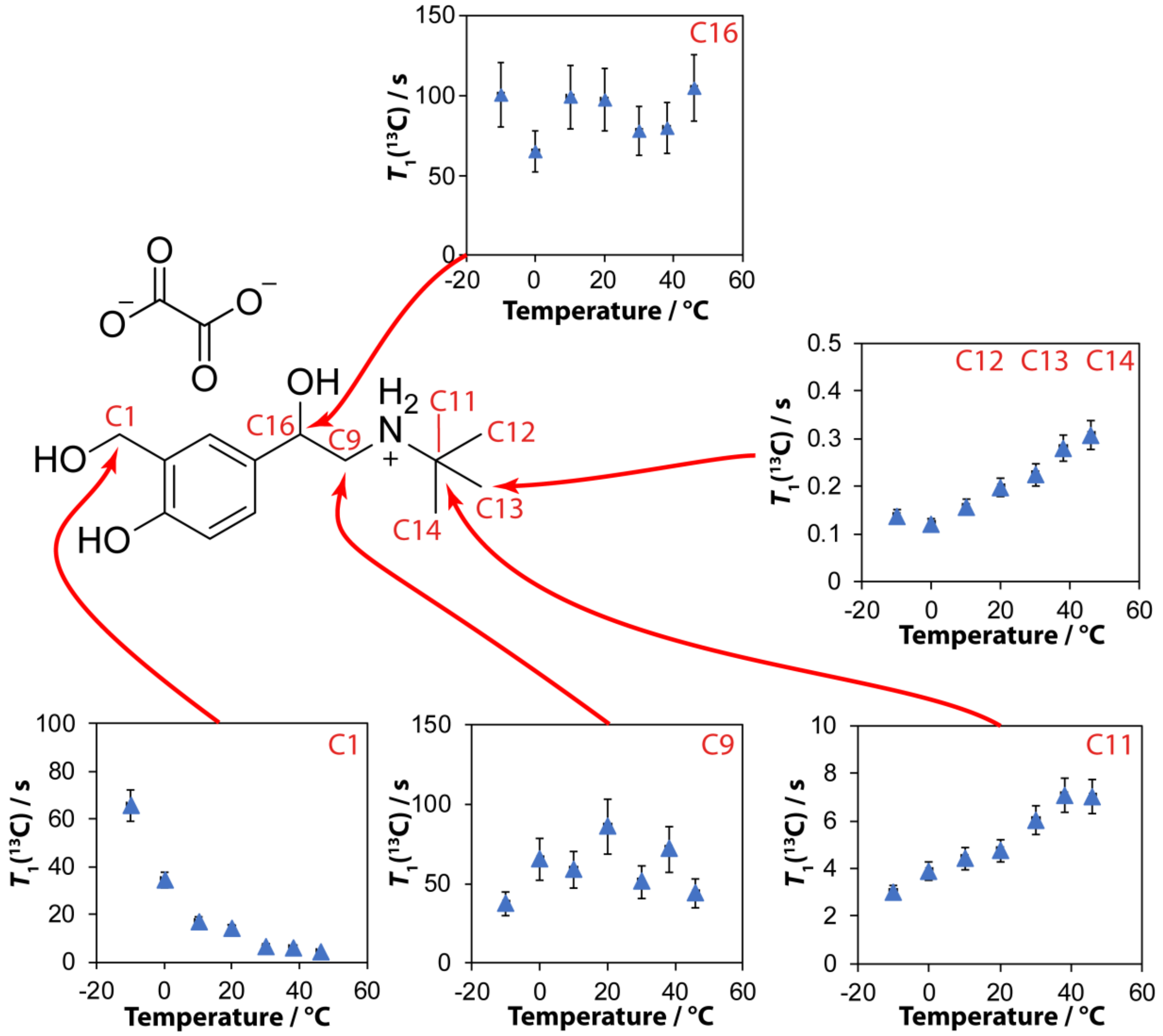

Figure S18. Experimental ^{13}C spin-lattice relaxation times, $T_1(^{13}C)$, as a function of the temperature for sample **1a**.

Table S9. Experimental ^{1}H $T_{1\rho}$ relaxation times for selected peaks in sample **1a** (see Figure S20).

	^{1}H $T_{1\rho}$ / ms		
phase	C4	C11	C9
major	2.9 ± 0.2	2.5 ± 0.2	2.7 ± 0.2
minor	2.6 ± 0.1	2.3 ± 0.2	2.3 ± 0.2

[1] The ^{1}H $T_{1\rho}$ experiments were performed at 11.7 T ($\nu_L(^{13}C)$ = 125.8 MHz, ν_{MAS} = 12.5 kHz) using a π/2 (^{1}H) $\tau_{spin\text{-}lock}$ CP t_{acq} pulse sequence. The experiment was performed using a 4 mm MAS probe, acquiring 1024 transients with a recycle delay of 3 s. A total of 8 increments were acquired with spin-lock times of: 0 ms, 1 ms, 2 ms, 5 ms, 7 ms, 10 ms, 12 ms, and 15 ms.

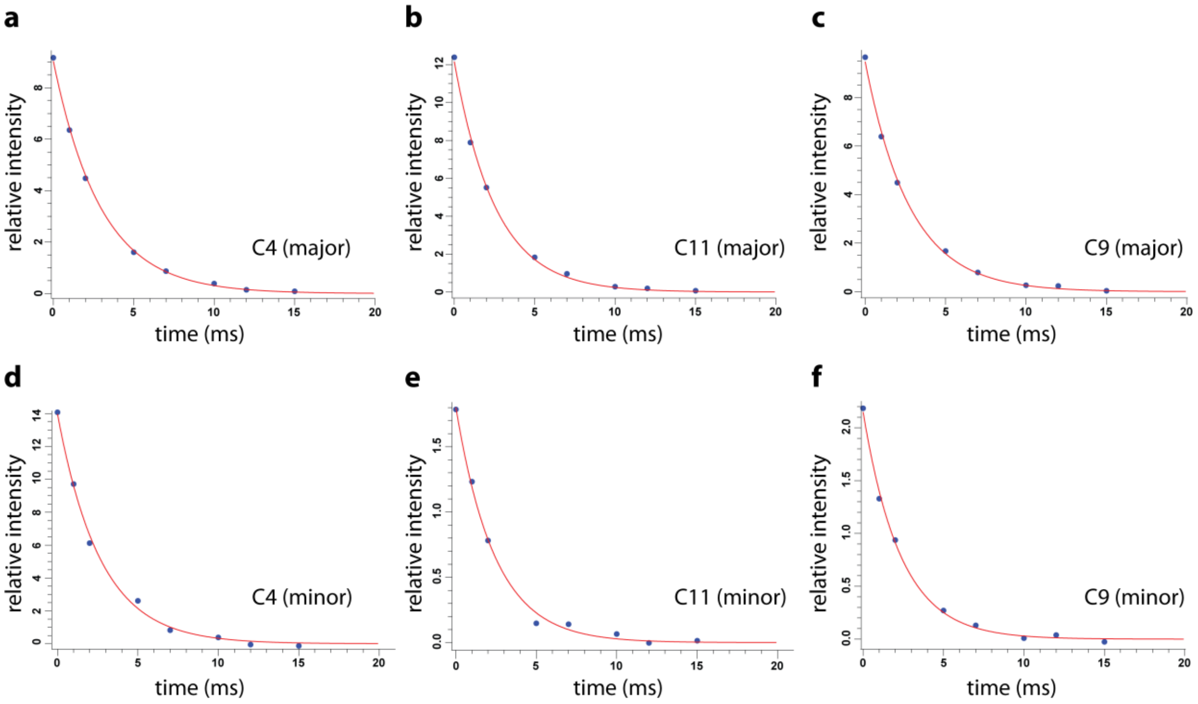

Figure S19. Fits of experimental $T_{1\rho}(^1\text{H})$ data to a decaying exponential function as detected at specific ^{13}C resonances: (a) C4 (major), (b) C11 (major), (c) C9 (major), (d) C4 (minor), (e) C11 (minor), and (f) C9 (minor). The $T_{1\rho}(^1\text{H})$ values are presented in Table S9. The ^{1}H $T_{1\rho}$ experiments were performed at 11.7 T ($\nu_L(^{13}\text{C})$ = 125.8 MHz, ν_{MAS} = 12.5 kHz) using a $\pi/2$ (^{1}H) $\tau_{\text{spin-lock}}$ CP t_{acq} pulse sequence. The experiment was performed using a 4 mm MAS probe, acquiring 1024 transients with a recycle delay of 3 s.

Section S6. X-ray diffraction data for all single crystals analysed of salbutamol oxalate

Table S10. Crystal data and structure refinement for salbutamol oxalate, sample **1a**.

Trial	#1	#2	#3
Empirical formula	$C_{14}H_{22}NO_5$	$C_{14}H_{22}NO_5$	$C_{14}H_{22}NO_5$
Temperature (K)	150.00 (10)	150.00 (10)	150.00 (10)
Crystal system	monoclinic	monoclinic	monoclinic
Space group	$P2_1/n$	$P2_1/n$	$P2_1/n$
a (Å)	8.40511 (19)	8.3986 (3)	8.4075 (4)
b (Å)	6.20065 (14)	6.20228 (16)	6.2047 (2)
c (Å)	27.6803 (6)	27.6418 (8)	27.6621 (12)
α (°)	90	90	90
β (°)	97.211 (2)	97.154 (3)	97.178 (4)
γ (°)	90	90	90
Volume (Å)	1431.21 (6)	1428.66 (7)	1431.71 (10)
Z	4	4	4
ρ_{calc} (g/cm^3)	1.320	1.319	1.319
μ (mm^{-1})	0.829	0.830	0.829
F(000)	612.0	610.0	612.0
Radiation	Cu Kα (1.54184 Å)	Cu Kα (1.54184 Å)	Cu Kα (1.54184 Å)
2θ range for data collection (°)	10.694 to 147.296	10.704 to 147.144	10.692 to 145.99
Reflections collected	7324	7104	7435
Goodness-of-fit on F^2	1.029	1.074	1.041
$R_{\text{all data}}$	R_1= 0.0560<br>WR_2= 0.1232	R_1= 0.0513<br>WR_2= 0.1173	R_1= 0.0622<br>WR_2= 0.01399
R [I>=2θ(I)]	R_1= 0.0469<br>WR_2= 0.1164	R_1= 0.0444<br>WR_2= 0.1117	R_1= 0.0521<br>WR_2= 0.1316
Largest diff. peak/hole (e Å^{-3})	0.35 / –0.31	0.33/ –0.26	0.36/ –0.26
Chemical occupancy [a] (*C-OH)	0.161 : 0.839(6)	0.121 : 0.879(5)	0.132 : 0.868(6)
CSD deposition No.	2106949	21069452	21069450

[a] The standard uncertainties were noted down before site occupancies were fixed.

Table S11. Crystal data and structure refinement for salbutamol oxalate, sample **1b**.

Trial	#1	#2	#3
Empirical formula	$C_{14}H_{22}NO_5$	$C_{14}H_{22}NO_5$	$C_{14}H_{22}NO_5$
Temperature (K)	150.00 (10)	150.01 (10)	150.00 (10)
Crystal system	monoclinic	monoclinic	monoclinic
Space group	$P2_1/n$	$P2_1/n$	$P2_1/n$
a (Å)	8.40087(19)	8.4010 (3)	8.4008(3)
b (Å)	6.20198(16)	6.2001 (2)	6.2030(2)
c (Å)	27.6590(5)	27.6519 (9)	27.6530(9)
α (°)	90	90	90
β (°)	97.195(2)	97.211(3)	97.228(3)
γ (°)	90	90	90
Volume (Å)	1429.74(6)	1428.90(8)	1429.55(8)
Z	4	4	4
ρ_{calc} (g/cm^3)	1.321	1.323	1.321
μ (mm^{-1})	0.830	0.832	0.830
F(000)	612.0	613.0	612.0
Radiation	Cu Kα (1.54184 Å)	Cu Kα (1.54184 Å)	Cu Kα (1.54184 Å)
2θ range for data collection (°)	10.7 to 147.414	10.7 to 147.512	6.444 to 146.134
Reflections collected	7863	7518	8406
Goodness-of-fit on F^2	1.065	1.046	1.048
$R_{\text{all data}}$	R_1= 0.0447<br>WR_2= 0.1063	R_1= 0.0532<br>WR_2= 0.1192	R_1= 0.0556<br>WR_2= 0.1160
R [I>=2θ(I)]	R_1= 0.0415<br>WR_2= 0.1036	R_1= 0.0459<br>WR_2= 0.1131	R_1= 0.0448<br>WR_2= 0.1091
Largest diff. peak/hole (e Å^{-3})	0.30/ –0.33	0.30/ –0.23	0.29/ –0.24
Chemical occupancy [a] (*C-OH)	0.150 : 0.850(4)	0.140 : 0.860(5)	0.129: 0.871(5)
CSD deposition No.	21069460	21069453	21069456

[a] The standard uncertainties were noted down before site occupancies were fixed.

Table S12. Crystal data and structure refinement for salbutamol oxalate, sample **1c**.

Trial	#1	#2	#3
Empirical formula	$C_{14}H_{22}NO_5$	$C_{14}H_{22}NO_5$	$C_{14}H_{22}NO_5$
Temperature (K)	150.00 (10)	150.01 (10)	150.00 (10)
Crystal system	monoclinic	monoclinic	monoclinic
Space group	$P2_1/n$	$P2_1/n$	$P2_1/n$
a (Å)	8.4069(2)	8.40076(19)	8.4006(4)
b (Å)	6.20466(16)	6.20553(14)	6.2011(3)
c (Å)	27.6775(8)	27.6588(6)	27.6450(14)
α (°)	90	90	90
β (°)	97.215(3)	97.177(2)	97.178(4)
γ (°)	90	90	90
Volume (Å)	1432.29(7)	1430.59(5)	1428.83(12)
Z	4	4	4
ρ_{calc} (g/cm^3)	1.319	1.320	1.322
μ (mm^{-1})	0.828	0.829	0.830
F(000)	612.0	612.0	612.0
Radiation	Cu Kα (1.54184 Å)	Cu Kα (1.54184 Å)	Cu Kα (1.54184 Å)
2θ range for data collection (°)	10.692 to 146.782	10.7 to 147.116	10.702 to 147.326
Reflections collected	7492	7982	7289
Goodness-of-fit on F^2	1.040	1.066	1.071
$R_{\text{all data}}$	R_1= 0.0501<br>WR_2= 0.1099	R_1= 0.0412<br>WR_2= 0.0975	R_1= 0.0633<br>WR_2= 0.1297
R [I>=2θ(I)]	R_1= 0.0439<br>WR_2= 0.1055	R_1= 0.0386<br>WR_2= 0.0979	R_1= 0.0514<br>WR_2= 0.1206
Largest diff. peak/hole (e Å^{-3})	0.35/ –0.29	0.35/ –0.30	0.37/ –0.29
Chemical occupancy [a] (*C-OH)	0.153 : 0.847(5)	0.148: 0.852(4)	0.165: 0.835(6)
CSD deposition No.	21069455	21069459	21069457

[a] The standard uncertainties were noted down before site occupancies were fixed.

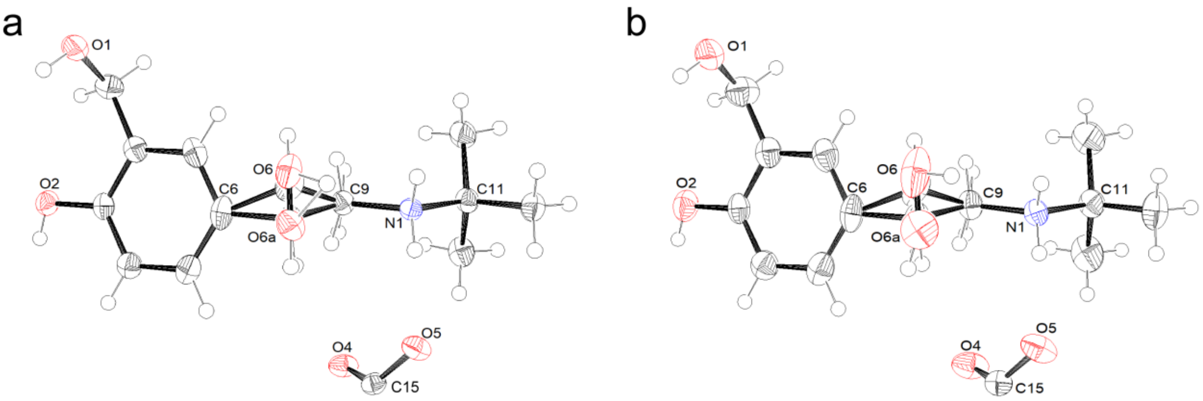

Figure S20. ORTEP-3[3] plots of structure **1b** acquired at (a) 150 K and (b) 298 K.

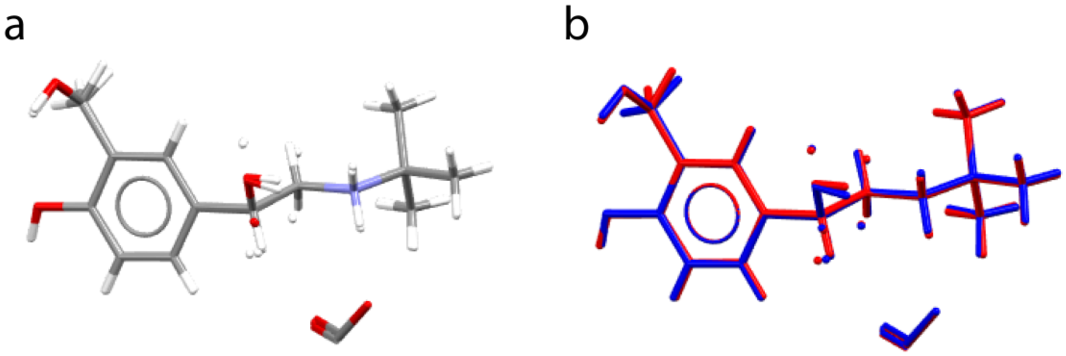

Figure S21. Overlay of structure **1b** acquired at 150 K and 298 K. The structure acquired at 150 K is represented in blue, whereas the structure acquired at 298 K is represented in red. The RMSD1 between both structures is 0.032 Å.

Table S13. Comparison of the crystal densities obtained from single-crystal XRD for several salbutamol compounds.

compound	acquisition temperature	density	reference
salbutamol sulfate	RT	1.321 g cm^{-3}	SALBUT, Ref 89 in the main text
salbutamol sulfate monohydrate	110 K	1.336 g cm^{-3}	JOSKUZ, Ref 85 in the main text
salbutamol benzoate	RT	1.236 g cm^{-3}	TEGJOH, Ref 109 in the main text
salbutamol oxalate at room 289 K	298 K	1.299 g cm^{-3}	this work
salbutamol oxalate at 150 K	150 K	1.322 g cm^{-3}	this work

References.

(1) Leger, J. M.; Goursolle, M.; Gadret, M.; Carpy, A. Structure cristalline du sulfate de salbutamol [tert-butylamino-2 (hydroxy-4 hydroxymethyl-3 phenyl)-1 ethanol.0.5H2SO4]. *Acta Cryst. B* **1978**, *34* (4), 1203-1208. DOI: doi:10.1107/S056774087800521X.
(2) Salager, E.; Day, G. M.; Stein, R. S.; Pickard, C. J.; Elena, B.; Emsley, L. Powder crystallography by combined crystal structure prediction and high-resolution ^{1}H solid-state NMR spectroscopy. *J. Am. Chem. Soc.* **2010**, *132* (8), 2564-2566. DOI: 10.1021/ja909449k.
(3) Farrugia, L. J. WinGX and ORTEP for Windows: an update. *J. Appl. Crystallogr.* **2012**, *45* (4), 849-854. DOI: 10.1107/s0021889812029111.
